# Bronze Age innovations and impact on human diet: A multi-isotopic and multi-proxy study of western Switzerland

**DOI:** 10.1371/journal.pone.0245726

**Published:** 2021-01-27

**Authors:** Alessandra Varalli, Jocelyne Desideri, Mireille David-Elbiali, Gwenaëlle Goude, Matthieu Honegger, Marie Besse

**Affiliations:** 1 Laboratory of Prehistoric Archaeology and Anthropology, Department F.-A. Forel, Section of Earth and Environmental Sciences, University of Geneva, Geneva, Switzerland; 2 Aix Marseille Univ, CNRS, Minist of Culture, LAMPEA, Aix-en-Provence, France; 3 Institut d’Archéologie, University of Neuchâtel, Hauterive, Switzerland; University of Pisa, ITALY

## Abstract

The archaeological Bronze Age record in Europe reveals unprecedented changes in subsistence strategies due to innovative farming techniques and new crop cultivation. Increasing cultural exchanges affected the economic system. The inhabitants of Switzerland played a pivotal role in this European context through relationships with the Mediterranean, the High and Middle Danube regions and the Alps thanks to the area’s central position. This research aims to reconstruct, for the first time in Switzerland, human socio-economic systems through the study of human diet, herding and farming practices and their changes throughout the Bronze Age (2200–800 BCE) by means of biochemical markers. The study includes 41 human, 22 terrestrial and aquatic animal specimens and 30 charred seeds and chaff samples from sites in western Switzerland. Stable isotope analyses were performed on cereal and legume seeds (δ^13^C, δ^15^N), animal bone collagen (δ^13^C_coll_, δ^15^N, δ^34^S), human bone and tooth dentine collagen (δ^13^C_coll_, δ^15^N,) and human tooth enamel (δ^13^C_enamel_). The isotopic data suggest a) an intensification of soil fertilization and no hydric stress throughout the Bronze Age, b) a human diet mainly composed of terrestrial resources despite the proximity of Lake Geneva and the Rhone river, c) a diet based on C_3_ plants during the Early and Middle Bronze Age as opposed to the significant consumption of ^13^C-enriched resources (probably millet) by individuals from the Final Bronze Age, d) no important changes in dietary patterns throughout an individual’s lifespan but a more varied diet in childhood compared to adulthood, e) no differences in diet according to biological criteria (age, sex) or funerary behavior (burial architecture, grave goods).

## Introduction

The Bronze Age (2200–800 BCE) is a period of important social and economic growth. Large cultural and ethnic traditions, with distinctive features, arose in Europe for the first time [1], developing in a larger, connected framework. This study explores the biochemical aspects of this phase of economic and social changes, which influenced every aspect of human life, including lifestyle, subsistence strategies and food habits. The reconstruction of farming practices, land use and, consequently, dietary behaviors, which directly affect prehistoric communities’ way of life, contribute to the understanding of these complex socio-economic dynamics.

Archaeological evidence suggests that Bronze Age communities lived on agriculture, animal husbandry and wild plant harvesting [2, 3]. From the Bronze Age onwards, the south of central Europe was characterized by the expansion of wide open spaces, reducing the areas covered by permanent forests [4]. These open areas were cultivated or used as pasture for livestock. The spread of the cultivated land led to the exploitation of less fertile soil, especially during the Final Bronze Age, a time of demographic growth [5]. To increase agricultural production, significant innovations were introduced and a number of new crops were cultivated, adding to the diversity of food resources [2], especially in areas characterized by poor soils or during periods of drought [6]. New management systems like crop rotation and the use of manure as well as new farming strategies which involved controlling the hydric systems developed, leading to a more intense and rationalized agricultural activity [7, 8].

The Bronze Age was also characterized by the increased mobility of goods and people, in long-distance exchange networks. Indeed, one of the main driving forces at the origin of large cultural complexes was the exchange of metal and other raw materials across Europe from only a few key areas and, consequently, long-distance trade transport [9, 10]. The distribution of bronze metallurgy, which requires both copper and tin, uncommon and unevenly distributed ore deposits, led to heightened relationships at both the local and regional levels and to the multiplication of exchanges at the European level [11–13]. Indeed, a new globalized world of interconnectivity took shape and the movement of people was at the base of this phenomenon, as has been confirmed by aDNA research [14–16].

In this context of greater European networking and socio-economic development, the archaeological record shows that Switzerland played a major role because of its strategic position for exchanges with both central and southern Europe [12, 17, 18]. Swiss archaeological sites from this period showcase a mixture of local traditions and influences from surrounding societies [18, 19]. Consequently, Switzerland is an exceptional observatory for the reconstruction of dietary patterns, and subsistence strategies during the Bronze Age. The region is characterized by high environmental variability: the rapidly changing biotopes have different natural resources, making this territory even more interesting for the study of subsistence strategies.

The aim of this research was to investigate how dietary habits and farming practices changed during the Bronze Age through a multi-isotopic approach on human, animal and botanical remains. Isotopic analyses accomplished on the entire trophic chain can clarify a) how agricultural practices evolved over time, b) whether manuring occurred or not, c) crops hydric condition and d) changes in dietary patterns. Some large European necropoles of the Bronze Age, such as Arano di Cellore, Olmo di Nogara (Italy) [20–22] and Singen-am-Hohentwiel (Germany) [23], have already been the subject of dietary investigations providing key economic details about agricultural practices. The high dietary variability detected in biochemical studies of the Bronze Age of southern Europe [20, 21, 24, 25] has led to further research on the spread of new plant foodstuffs, like C_4_ plants, in Europe from the Middle Bronze Age. Switzerland, particularly its western area, offers excellent archaeologically and chronologically well-documented datasets to explore these types of questions through a multi-isotopic and multi-proxy approach.

### The Bronze Age of western Switzerland

**Fig 1:**
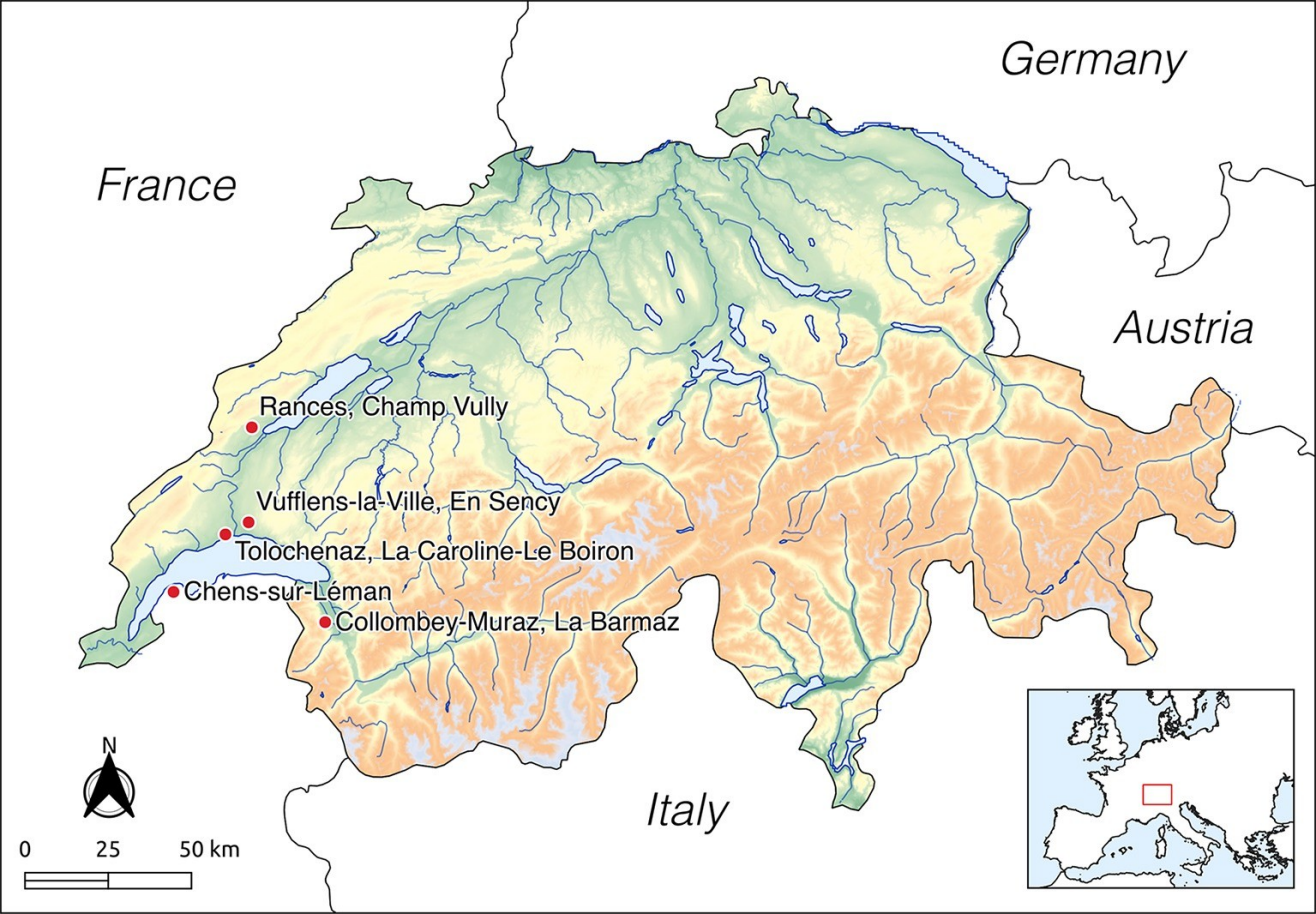
Location of the archaeological sites in western Switzerland included in this study (the map was made in QGIS 3.16 using Natural Earth, GMTED2010 and DIVA-GIS metadata publically available).

Placed in the north-western portion of the Alpine mountain chain, Switzerland has a strategic geographic position. It is crossed by a large plateau oriented northeast-southwest, with a rich hydrographic network and several lakes, connecting western and central Europe. In addition, several Alpine passages provide access to southern Europe through northern Italy (Fig 1). At the crossroads of various exchange networks and cultural influences, the Bronze Age of Switzerland (Early Bronze Age: 2200–1500 cal. BCE, Middle Bronze Age: 1500–1300 cal. BCE, Recent and Final Bronze Age: 1300–800 cal. BCE [19]) is characterized by a mosaic of cultural groups, both in time and space (e.g., culture of the Valais [18, 26]). However, current knowledge of this cultural variability is limited by the uneven quality of the archaeological record from the different chronological phases [27].

Despite these limitations, several lines of archaeological evidence point towards an increase in socio-political, economic and technological complexity throughout the Swiss Bronze Age. Funerary practices shifted from cemeteries of individual burials in the Early Bronze Age [28–30] to grouped graves under tumuli and rare burials with multiple individuals in the Middle Bronze Age [31–33] to mixed necropoles with individual cremations and inhumations at the end of the Bronze Age [34, 35]. Grave goods are present in some burials in all Bronze Age periods: they are limited to bronze artefacts in the Early Bronze Age, while from the Middle Bronze Age pottery occurs sometimes, becoming more frequent during the late Bronze Age. Metal artefacts continue to be present, especially prestige objects like in Middle Bronze Age burials [19, 36] and in the later Bronze Age ones [35]. The increasingly specialized practice of metallurgy required exchanges with the south of the Alps, the Danube Basin and the eastern Mediterranean [37–39].

This trajectory of increased complexity and social stratification is difficult to study archaeologically for the later Bronze Age periods because the practice of cremation was introduced in the Middle Bronze Age and became almost exclusive during the Recent and Final Bronze Age in Switzerland, as in most of Europe (e.g., [40]). Exceptions to this pattern are the Final Bronze Age cemeteries from the Lake Geneva Basin and the upper Rhone valley [35]. Indeed, in the Final Bronze Age, both cremations and inhumations were present in south-western Switzerland but cremations seemed more complex in terms of funerary architecture and grave goods, suggesting that they may have been reserved for the elite [35]. This contrasts with earlier periods in the same areas where the small size of the necropoles suggests the existence of small communities with limited social differences.

In parallel with these social and demographic changes, during the Bronze Age, subsistence patterns intensified, involving several aspects of the production economy. Throughout the Bronze Age, the economy was based on agriculture, cattle breeding and woodland exploitation, in substantial continuity with the agro-pastoral economies practiced since the Neolithic [41, 42]. However, in addition to wheat and barley, new varieties of pulses and cereals, such as lentils, beans, spelt and millet were introduced [42–45]. Not only the number of plants increased, but also the production techniques improved and land use changed [42, 46]. There is evidence for the use of crop rotation, fertilization and water supply systems to increase agricultural production [47]. Animal husbandry still played a crucial role in the economy, with an increased dominance of sheep and goats at the end of the Bronze Age, although the main meat supply was provided by cattle [48–50]. Sediment records and strontium isotope analysis performed on animal remains support seasonal movement of livestock to higher elevations [51]. Evidence for a well-organized use of the territory is demonstrated by the exploitation of wetlands around the lakes: wet meadows beyond the reedbeds, in all likelihood seasonally flooded, provided bedding for livestock.

All these increasingly complex agricultural and pastoral activities are apparent in the archaeological record [27, 41]. However, there remain important gaps in current knowledge, such as the timing and geographic patterning of the introduction of these new agricultural and pastoral techniques. In addition, research has focused on archaeobotanical and archaeozoological remains without any direct evaluation of the resources consumed through biochemical analyses. As a result, it is not known whether the new plants were destined for animal or human consumption, whether manuring was routinely applied or not and whether it was used on all crops or only on a select few. Similarly, almost no information is available about hydric conditions and if some crops were preferentially watered compared to others. Multi-proxy isotopic studies on well-dated material have the potential to significantly improve our understanding of Bronze Age economic intensification, changes to human dietary patterns and mobility events.

### The cemeteries

Collombey-Muraz, La Barmaz is located in the Valaisan Chablais, around 15 kms from the oriental limit of Lake Geneva (Fig 1). The site has Neolithic and Bronze Age occupations and is composed of the cemeteries of La Barmaz I and II and the settlement of the Refuge hill [18, 30]. The Early Bronze Age necropolis of La Barmaz I (2200–1550 cal. BCE) includes 15 individual burials. Anthropological analyses identified 13 adults of both sexes and two young juveniles (< 5 years old) [30] (S1 Fig). The present study includes only the 13 adults. Seven graves contained exclusively bronze objects as grave goods: awls, spiraled rings, pearls, a double-spiral pendant, a dagger and an axe [18]. The funerary architecture, the ritual and the grave goods do not suggest any social differences between the individuals [30].

The necropolis of Vufflens-la-Ville, En Sency is located in the Venoge valley, about 9 kms northwest of Lausanne, on a small hill used as a gravel pit (Fig 1) [31, 52]. The necropolis was in use from the Middle Bronze Age to the Early Iron Age. A burial stone mound (tumulus) was erected at the beginning of the Middle Bronze Age, covering the central double burial of an elderly woman and a young man. Two other individual burials, one of an older male and the other of a juvenile, overlap with the edge of the burial mound and date to the Middle Bronze Age (S2 Fig). Contemporary with the latter two individual burials, there is a collective grave of four women and two adolescents in a pit on the south-western part of the tumulus (S2 Fig). The collective burial contained more than 80 amber beads, different pendants and a jug. Two male burials without grave goods located on the western part of the site are contemporary with the previous burials and two other cremated burials belong to later periods. In total, 12 Middle Bronze Age inhumations were found [31]. The individual, double, and collective graves contain individuals of different ages and sex. Anthropological studies identified seven adults and five juveniles. The grave goods suggest that this small cemetery was likely reserved for a local elite [31].

The site of Tolochenaz, Le Boiron-La Caroline is situated to the north of Lake Geneva, in the commune of Tolochenaz (Fig 1). The cemetery is split between two lake terraces, ten and 30 meters high each, Le Boiron and La Caroline, both dated to the Final Bronze Age (1050–800 cal. BCE). Tolochenaz was a bi-ritual cemetery, where both inhumation and cremation were performed. Le Boiron was discovered at the beginning of the 19^th^ century and even if more than 70 graves were initially discovered, more recently only 17 burials and 15 cremations have been recorded [34]. In 2009, the exploitation of a gravel pit on the 30-meter high terrace led to the discovery of 20 graves in the La Caroline area [35]: 19 inhumations, mainly in monolithic coffins, and one cremation. One individual (LaC4) differs from the rest for his unique funerary structure. He was buried prone in a straight ditch surrounded by a circular ditch, while all the other inhumations were supine with extended lower limbs in rectangular pits. Several graves delivered funeral artefacts such as an awl, pins, ankle rings, bracelets, rings, amber and glass beads, razors and ceramic vessels (S3 Fig). For this study, ten individuals from La Caroline and six from Le Boiron were included, for a total of 12 adults of both sexes and four juveniles.

## Stable isotope analyses to reconstruct human dietary habits

Carbon isotopic ratios allow us to distinguish the environment where humans acquired their resources (e.g., terrestrial vs. aquatic) and the type of plants consumed. The C_3_ type plants are typical of a temperate environment (barley, wheat) and they prevail in Switzerland and in all of Europe, whilst C_4_ type plants usually reflect an open and warm environment and have higher values (millet, sorghum) [53–55]. The C_4_ plants most cultivated by prehistoric communities were millet varieties, mainly *Panicum miliaceum* and *Setaria italica* [20, 21]. Thus, the possibility of detecting C_4_ plant intake is particularly relevant to the present study as it may help identify the first C_4_ consumers in central Europe. The isotopic ratios of nitrogen are used to determine the trophic level occupied within the food chain. Plants, being at the base of the food chain, present lower δ^15^N values and as a consequence the δ^15^N values of predators are higher than that of their prey [56, 57]. Finally, isotopic ratios of sulfur help identify the origin of dietary resources (terrestrial, aquatic and marine) and detect the presence of non-local individuals [24, 58–60].

From one consecutive link of the chain to the next, a fractionation in favor of the heavy isotope occurs. The concentration in heavy isotope increases (^13^C, ^15^N, ^34^S) and the isotope ratios rise at a rate estimated from 0 to 1‰ for carbon, from 3 to 5‰ for nitrogen, and lower than 1‰ for sulfur [56, 57, 61–63].

By analyzing remains from different sources, the isotopic values for each source are defined.

Archaeobotanical studies increasingly refer to experimental studies of traditional agricultural systems [64]. Even though environmental factors [54, 65–67] as well as geographical conditions [68] influence plants’ isotopic values, experimental investigations contribute to quantify the impact of anthropic activities on isotopic values, such as the use of manure on agricultural production [69, 70] and the hydric growing conditions [71–73].

Through the isotopic analyses of animal remains, the local isotopic variability of a diet can be determined. When considering wild and domestic species, environmental factors and husbandry practices can be taken into account as these can strongly influence the isotopic values of domestic animals [74]. Moreover, based on herbivore data, it is possible to evaluate the isotopic value of the fodder they consumed. As such, the estimated isotopic ratios from fodder provide a sample of the local vegetation, to which the values for cultivated plants can be compared [75]. Consequently, agricultural practices, such as the use of manure or irrigation systems for cultivated plants, can be evaluated.

The analyses of the human remains were conducted at both the individual and population level. By taking into account bone and teeth, the changes in diet over an individual’s lifespan can be traced because bone and teeth have different growth patterns and turnovers [76, 77]. They are both constituted of a mineral, bioapatite, and an organic component, collagen. On the one hand, bone collagen records the protein components of the diet, and its chemical composition reflects the food consumed over the last ca. 15 years of an adult’s life [78]. Teeth, on the other hand, record the biogeochemical information of childhood, which can vary between teeth because of their staged development. Finally, dentine collagen provides information on the protein intake at the beginning of an individual’s life. In addition to the study of protein intake, it is also possible to study the global energy provided through food (carbohydrates, lipids and proteins not used for protein tissue synthesis) by analyzing tooth enamel carbonates [79–81]. The δ^13^C value from apatite is dependent on the energy substrate because it is obtained from blood plasma CO_2_ which mainly comes from metabolizing carbohydrates. Consequently, the δ^13^C from apatite provides information on food sources consumed that are low on protein content (e.g. cereals), which are difficult to identify when different dietary resources overlap [79]. Based on experimental studies [82–84], the spacing between diet and bone apatite has been estimated to 10.7‰ ± 1.4‰ on average (mean value) and between diet and enamel apatite to ca. 13.4‰ ± 1.0‰ on average for a C_3_ and a C_3_-C_4_ diet [85]. Hence, through the carbon apatite–collagen spacing (Δ^13^C_ap–coll_) it is possible to distinguish the contribution of low (wide spacing) and high (small spacing) trophic level resources [86–88].

### Materials

Archaeological wild and domestic terrestrial and aquatic animals, as well as archaeobotanical specimens were sampled from the same areas where the humans lived and shown in Fig 1.

There are 41 humans in total: 13 from La Barmaz, 12 from Vufflens and 16 from Tolochenaz. Sex and age-at-death were analyzed previously (Table 1) [30, 31, 35]. Sex was determined using standards outlined for the cranium and the coxal bones [89–91]. Age-at-death was assessed using criteria proposed by [92, 93] for adults and by [77, 94–96] for juveniles. To limit the uncertainties linked with using different methods, individuals were grouped into four age categories (Table 1). Collagen was extracted from cortical bone and dentine from the crown of the second molar, except for five individuals for whom samples were taken from the crown of a canine, a premolar, a first molar and two incisors.

**Table 1:** Human remains analyzed with their relevant anthropological and archaeological information.

Sites	ID	Burial	Grave goods	Skeletal element	Tooth	Sex	Age	Chronological period^b^	Date cal BCE (2σ)	Date BP
**Collombey-Muraz, La Barmaz**	BA3	N3	P	ulna R	M_2_ R	F	A	EBA (BzA2)		
	BA5	N5	A	ulna L	M_2_ R	M	A	EBA		
	BA6a/b	N6a/N6b	nd	ulna L	M_2_ R	F	A	EBA		
	BA6	N6	P	ulna L	M_2_ R	F	A	EBA (BzA2b,c)		
	BA22	N22	P	ulna L	M_2_ R	M	A	EBA (BzA2)		
	BA23	N23	A	tibia R	M_2_ R	M	A	EBA		
	BA25	N25	A	ulna L	M_2_ R	M	A	EBA		
	BA26	N26	A	ulna L	M_2_ R	M	A	EBA		
	BA28	N28	A	humerus R	M^2^ R	F	A	EBA		
	BA42	N42	P	ulna L	M_2_ R	M	YA	EBA (BzA2b)		
	BA50	T50	A	ulna L	M_2_ R	F	A	EBA	1618–1224 cal BCE	CRG 1339: 3172±82 BP
	BA53	N53	A	tibia R	-	M	A	EBA		
	ZHB	ZHB	P	ulna L	M_2_ R	M	A	EBA (BzA2a)		
**Vufflens-la-Ville, En Sency**	VF1	VF 94/st 1 ind 1	P	ulna R	M_2_ R	F	A	MBA (recent BzB)		
	VF2	VF 94/st 1 ind 2	P	fibula	M_2_ R	M	YA	MBA (recent BzB)	1690–1430 cal BCE	ETH-15757: 3285±65 BP
	VF3	VF 95/st 4^**b**^ ind 1	P	humerus R	M_2_ L	F	A	MBA (BzC)		
	VF4	VF 95/st 4^**b**^ ind 2	P?	humerus L	M_2_ R	I	C	MBA (BzC)	1690–1370 cal BCE	ETH-17761: 3235±80 BP
	VF5	VF 95/st 4^**b**^ ind 3	P?	ulna R	M^2^ L	F	YA	MBA (BzC)		
	VF6	VF 95/st 4^**b**^ ind 4	nd	ulna L	C sup R	I	C	MBA (BzC)		
	VF7	VF 95/st 4^**b**^ ind 5	P	ulna R	M_2_ R	F	a	MBA (BzC)		
	VF8	VF 95/st 4^**b**^ ind 6	P	ulna L	M_2_ L	F	A	MBA (BzC)	1520–1250 cal BCE	ETH-15758: 3120 ± 60 BP
	VF9	VF 95/st 9	P	radius L	M_2_ L	M	A	MBA (BzB/C)	1530–1190 cal BCE	ETH-17758: 3130 ± 75 BP
	VF10	VF 95/st 10	P	radius	M_2_ L	I	C	MBA (BzB/C)	1520–1260 cal BCE	ETH-15759: 3125 ± 55 BP
	VF11	VF 95/st 11	A	radius L	M2 R	M	a	MBA	1880–1500 cal BCE	ETH-17759: 3370 ± 70 BP
	VF12	VF 95/st 14	A	ulna R	M_2_ L	M	YA	MBA	1880–1510 cal BCE	ETH-17760: 3380 ± 70 BP
**Tolochenaz, Le Boiron, La Caroline**	LaC1	st. 1052	P	radius R	M2 L	M	A	FBA (HaB3)		
	LaC2	st. 1061	P	radius R	M2 R	F	A	FBA (early HaB1)		
	LaC3	st. 1074	P	tibia R	M1 L	I	A	FBA (HaB2)		
	LaC4	st. 1111	P	ulna L	PM1 L	M (?)	A	FBA (recent HaB3)		
	LaC5	st. 1057	A	ulna R	M2 R	I	C	FBA (HaB)		
	LaC6	st. 1080	P	humerus	I2 R	I	C	FBA (HaB3)		
	LaC7	st. 1018	P	clavicle R	I2 R	I	C	FBA (HaB1*)		
	LaC8	st. 1070	P	femur L	-	I	C	FBA (HaB)		
	LaC9	st. 1083	P	tibia R	-	I	A	FBA (HaB3)		
	LaC10	st. 1071	P	femur R	-	I	A	FBA (HaB3)		
	LeB1	tb XXIV	nd	femur L	M2 L			FBA (?)		
	LeB2	tb IX-X	P	femur R	-	M		FBA (early HaB3)		
	LeB3		nd	femur R	-			FBA		
	LeB4	tb I	nd	femur R	M2 R			FBA		
	LeB5	tb III	P	femur L	M2 L			FBA (recent HaB3)		
	LeB6		nd	humerus L	M2 R			FBA		

^a^The chronology is according to [13, 97, 98].

^b^VF 95/st 4: presence of grave goods that could not be attributed to a specific individual.

Abbreviations: R = right; L = left; child (C) = 5–15 yrs; adolescent (a) = 16–19 yrs; young adult (YA) = 20–29 yrs; adult (A) > 30 yrs; M = male; F = female; I = indeterminate; EBA = Early Bronze Age; MBA = Middle Bronze Age; FBA = Final Bronze Age; Grave goods: P = present, A = absent, ND = not defined.

The terrestrial animals date to the Early (La Barmaz), Middle (Rances, Champ Vully) and Final Bronze Age (Chens-sur-Léman, Tougues). They include domestic and wild animals (*Ovis aries*, *Capra hircus*, *Bos Taurus*, *Sus domesticus*, *Cervus elaphus*) for a total of 30 individuals. Four samples of pike (*Esox lucius*) were sampled from Chindrieux (Lake of Bourget, Savoie), dating to the Final Bronze Age (Table A in S1 Text).

In addition, 30 seeds and chaff from cultivated plants from Chens-sur-Léman (Pré d’Ancy and Vereître) were analyzed (Table A in S1 Text). They are dated to the Early, Middle and Final Bronze Age in order to highlight any possible changes in agricultural practices across time. The C_3_ plant samples include hulled barley (*Hordeum vulgare*), emmer and einkorn wheat (*Triticum dicoccum*, *Triticum monococcum*), and pulses, such as beans (*Vicia faba*), for a total of 25 samples. The C_4_ plant samples include five seeds of broomcorn and foxtail millet (*Panicum miliaceum*, *Setaria italica*). Each isotopic measurement corresponds to the analysis of one seed.

## Methods

La Barmaz humans are in storage at the University of Geneva and animals are in the deposit of the Office for Archaeological Research of Valais; sampling permits were issued by the Office for Archaeological Research of Valais. Vufflens-la-Ville humans are stored in the Cantonal Museum of Archaeology and History of Lausanne and Tolochenaz humans in the store of Archeodunum SA and sampling permits were granted by the Cantonal Museum of Archaeology and History of Lausanne. Animals from Tougues and Chindrieux are stored in the deposit of the DRAC Auvergne-Rhône-Alpes Regional Archaeology Service and Rances animals are in the Natural History Museum of Geneva and the DRAC Auvergne-Rhône-Alpes provided the sampling authorizations. The botanical remains are stored in the Archaeological Research Center of Clermont-Ferrand and study permission was provided by the National Institute for Preventive Archaeological Research (INRAP).

Samples were prepared at the LAMPEA (UMR 7269, Aix-en-Provence, France) and the EA-IRMS analyses were performed at the Iso-Analytical Limited Laboratory (Cheshire, UK). The seeds and chaff were analyzed following the acid-base-acid protocol [69]. The samples were soaked in HCl (0.5 M, 70°C, for 30–60 min), then rinsed three times with distilled water and soaked in NaOH (0.1 M, 70°C, 60 min) and rinsed again and soaked in HCl (0.5 M, 70°C, for 30–60 min). Once dried in the oven, the samples were finely powdered.

Bone and dentine collagen was extracted following [99] and modified by [100]. Approximately 0.5 g of cortical bone was cleaned by surface abrasion to remove superficial contaminants and demineralized (0.5 M HCl, 4°C, for 7–14 days). The samples were then rinsed several times with distilled water and gelatinized (pH 3, 75°C, for 48 h). The resulting solution was filtered using an Ezee™ filter and the supernatant was freeze-dried for 48 h. Similarly, for each selected tooth, half the crown was cut with a saw and the samples were demineralized (0.5 M HCl, 4°C, for ca. 20 days). Once the dentine was soft, the samples were rinsed with distilled water and gelatinized (pH 3, 75°C, for 48 h). The solution was then filtered with Ezee™ filter and the supernatant was freeze-dried for 48 h, as was done for the bone.

Tooth enamel carbonates was pre-treated following the protocol of [101] on enamel powder. The samples were placed in NaOCl (2%–3%, 0.1 ml/1 mg, 24 h), rinsed and soaked in CH3COOH to remove diagenetic carbonates (1 M, 1 h, 1 mL/1 mg), rinsed again in a centrifuge and dried (65°C, 12 h).

No consensual criteria exist to assess the preservation of charred seeds and chaff and whether or not the resultant values are reliable. Here, the criteria proposed by [102] for %C, %N and C:N ratio are considered. Taking into account the average offsets between uncarbonized crop seeds and those heated for four, eight or 24 h at 215, 230, 245 or 260°C, the δ^13^C and δ^15^N values of carbonized crop were corrected for the charring effect by subtracting 0.11‰ and 0.31‰, respectively, according to the recommendations of [103]. Concerning the three chaff samples of *Triticum*, no criteria to establish the reliability of the results exist. Following the suggestions of two experimental studies, considering the offset between cereal grain and rachis [70, 104], +2.4‰ in δ^15^N and +1.9‰ in δ^13^C were added to the wheat chaff values in order to make the seed and the chaff isotope results comparable.

Bone and dentine collagen preservation was checked according to the following criteria: the yield of extraction (≥ 1%), the percentages of C, N and S (%C ≥ 30%, %N ≥ 10% and %S ≥ 0.15%), and the atomic C:N, C:S and N:S ratio (2.9 < C:N < 3.6, C:S = 600 ± 300 and N:S = 200 ± 100) [105–108]. For the tooth enamel carbonates, no set rules exist to distinguish reliable apatite samples [109, 110] because the apatite carbonate preservation in archaeological samples is difficult to estimate [111]. Even though enamel can be subject to alteration in certain burial conditions [111, 112], enamel is less porous than bone, limiting the risk of diagenetic modifications induced by contamination and degradation [111, 113].

The international standards used for collagen and bulk plants are bovine liver (IA-R042), a mixture of ammonium sulfate (IA-R045) and beet sugar (IA-R005), and a mixture of sugar cane (IA-R006) and ammonium sulfate (IA-R046). For carbonate apatite, they are calcium carbonate (IA-R022), carbonatite (NBS-18), chalk (IA-R066), and Carrara marble (IAEA-CO-1). Measurement reproducibility of a repeat sample is below 0.1‰ for δ^13^C_coll_, δ^13^C_enamel_, δ^15^N and δ^34^S values.

To reconstruct the dietary scenarios, two offsets were used for carbon: (a) 4.8‰ between the δ^13^C values of the plants and the collagen of their consumers and (b) 0.8‰ between the carbon isotope values of the collagen of consumers of the previous trophic level [114]. For nitrogen isotope compositions, a spacing of 4.0‰ was considered between the diet and the consumer of two different trophic levels [114].

For data interpretation and statistical analyses, Microsoft Excel and R v.3.6.1 were used. Bayesian models were elaborated using the software FRUITS [115].

## Results

### Botanical data

Of the 30 botanical remains, only one barley seed (VR6) was excluded after pre-treatment because it did not have enough material for analysis. The %C, %N and the C:N atomic ratios for all the other charred seeds fit within the expected ranges [102], with the exception of the %C of VE9 which is lower than in the others (17.7%) (Table 2).

**Table 2:** Isotopic results of the archaeological plant remains from Chens-sur-Léman, Pré d’Ancy (PA) and Vereître (VE).

Id	Chronological period	Species	Sample	%N	δ^15^N_AIR_	%C	δ^13^C_V-PDB_	C/N	Δ^13^C^a^
PAbot1	MBA-FBA (MB II-FB I)	*Vicia faba*	seed	4.7	2.6	44.9	-23.1	11.2	17.0
PAbot2	MBA-FBA (MB II-FB I)	*Hordeum vulgare*	seed	2.5	3.0	57.8	-24.8	27.2	18.8
PAbot3	FBA (FB IIb-IIIa)	*Panicum miliaceum*	seed	3.6	5.5	51.7	-9.4	16.9	
PAbot4	MBA-FBA (BM II-FB I)	*Hordeum vulgare*	seed	2.0	3.6	61.0	-24.9	35.1	18.9
PAbot5	MBA-FBA (BM II-FB I)	*Hordeum vulgare*	seed	2.0	4.0	55.6	-24.2	31.9	18.2
PAbot6	MBA-FBA (MB II-FB I)	Fabaceae	seed	4.5	3.3	50.6	-15.3	13.1	9.0
PAbot7	MBA-FBA (MB II-FB I)	Fabaceae	seed	7.7	1.8	56.3	-23.9	8.5	17.9
PAbot8	FBA (FB IIb/IIIa)	Fabaceae	seed	6.3	1.1	53.7	-24.2	10.0	18.2
PAbot9	EBA	*Triticum* sp	seed	3.0	4.4	60.1	-27.0	23.5	21.2
PAbot10	EBA	*Triticum* sp	seed	3.0	3.7	60.6	-26.2	23.3	20.3
PAbot11	EBA	*Triticum* sp	seed	1.9	3.6	53.8	-23.9	32.4	18.0
VE1	FBA (FB IIb)	*Triticum dicoccum*	seed	1.7	2.1	48.7	-24.7	33.3	18.7
VE2	FBA (FB IIb)	*Triticum dicoccum*	seed	2.8	3.4	43.9	-23.6	18.5	17.5
VE3	FBA (FB IIa)	*Triticum dicoccum*	seed	1.9	6.3	34.4	-23.6	21.5	17.5
VE4	FBA (FB IIa)	*Triticum dicoccum*	seed	3.0	3.1	54.7	-24.4	21.5	18.4
VE5	FBA (Early Ha)	*Hordeum vulgare*	seed	6.1	2.2	62.1	-24.3	12.0	18.2
VE6	FBA (Early Ha)	*Hordeum vulgare*	seed	*Not enough material to be analysed*					
VE7	FBA (FB IIa)	*Panicum miliaceum*	seed	3.0	6.1	52.7	-10.8	20.4	
VE8	FBA (FB IIa)	*Vicia* sp	seed	5.6	2.8	61.6	-26.1	12.9	20.2
VE9	FBA (FB IIa)	*Setaria italica*	seed	1.1	6.4	17.7	-10.5	19.2	
VE10	FBA (Early Ha)	*Vicia* sp	seed	3.1	6.7	52.1	-24.1	19.6	18.0
VE11	FBA (Early Ha)	*Vicia* sp	seed	1.6	6.5	46.4	-24.5	34.0	18.5
VE12	FBA (Early Ha)	*Vicia* sp	seed	2.0	5.3	64.6	-26.5	37.1	20.5
VE13	FBA (FB IIa)	*Triticum monococcum/dicoccum*	chaff	0.8	4.6	62.1	-25.4	87.4	17.4
VE14	FBA (Early Ha)	*Triticum monococcum/dicoccum*	chaff	0.7	2.5	59.4	-25.8	100.4	17.7
VE15	FBA (FB IIa)	*Setaria/Panicum*	seed	3.0	5.4	61.0	-10.1	23.9	
VE16	FBA (FB IIa)	*Setaria/Panicum*	seed	2.7	5.3	61.8	-10.5	26.3	
VE17	FBA (FB IIa)	*Triticum* sp	seed	2.1	2.6	54.0	-23.5	29.4	17.4
VE18	FBA (FB IIa)	*Triticum* sp	seed	2.6	5.7	53.1	-24.5	24.1	18.4
VE19	FBA (FB IIa)	*Triticum* sp	chaff	0.5	2.7	60.1	-25.3	133.3	17.2

^a^Calculated on the corrected values (-0.11‰ to δ^13^CV-PDB of the charred seeds and +1.9‰ to δ^13^CV-PDB of the chaff) using AIRCO2_LOESS data calibrator [71].

**Fig 2:**
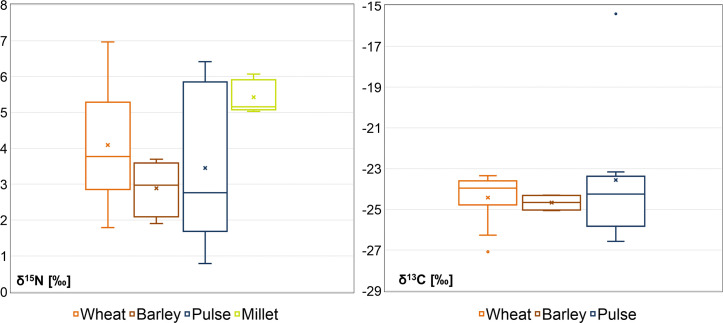
Box-plots showing the stable isotope results for wheat chaff and charred seeds. Corrected values have been used for a) δ^15^N results (-0.31‰ for the grains and +2.4‰ for the chaff to eliminate the charring effect) and b) δ^13^C results (-0.11‰ for the grains and +1.9‰ for the chaff). The boxes represent the first and third quartiles and the whiskers the maximum and minimum values, excluding the outliers. The continuous line illustrates the median and the cross represents the mean. For δ^13^C results only C_3_ plants are represented.

The isotopic ratios of the C_3_ plants range from 1.1‰ to 6.7‰ for δ^15^N (3.6‰ ± 1.5‰, n = 24) and from 27.0‰ to -15.3‰ for δ^13^C (-24.3‰ ± 2.2‰, n = 24). The C_4_ samples results range from 5.3‰ to 6.4‰ for δ^15^N (5.7‰ ± 0.5‰, n = 5) and from -10.8‰ to -9.4‰ for δ^13^C (-10.3‰ ± 0.5‰, n = 5). When pooling together all the samples of the same species in each of the different phases, no significant differences in N among species are detected, possibly due to the small sample size (approximate Kruskal-Wallis *p* = 0.103). In fact, the δ^15^N value of millet appears higher when compared to the other species. Significant differences by species are highlighted for δ^13^C (*p* = 0.001). When considering only C_3_ plants, no significant differences appear (*p* = 0.498) (Fig 2, Table 3).

**Table 3:** Statistical parameters for the botanical remains.

δ^15^N_AIR_^a^						δ^13^C_V-PDB_ ^b^				
species	n	min	max	Mean (1sd)	*p* (Kruskas Wallis)	min	max	Mean (1sd)	*p* (Kruskas Wallis)	*p* (Kruskas Wallis)
wheat	12	1.8	7.0	4.7 (1.6)	0.103	-27.1	-23.4	-24.4 (1.2)	***0*.*001***	0.498
barley	4	1.9	3.7	2.9 (0.8)		-24.9	-24.2	-24.7 (0.4)		
pulses	8	0.8	6.4	3.5 (2.1)		-26.5	-15.3	-23.6 (3.5)		
millet	4	5.0	6.1	5.4 (0.5)		-10.9	-9.6	-10.4 (0.5)		

^a^ Correction of -0.31‰ for δ^15^N_AIR_ to eliminate the effect of carbonization and +2.4‰ for chaff.

^b^ Correction of -0.11‰ to δ^13^C_V-PDB_ for the charred seeds and +1.9‰ for the chaff.

### Animal and human data

Of the 22 animal samples, 19 have a good yield. Collagen preservation and carbon, nitrogen and sulfur are present in enough quantity attesting to the reliability of the results. Two cattle (BANF6 and BANF7) with %N, %C and %S below the accepted values but very close to the accepted limits, have been included in the study but are considered with caution. Similarly, a pig (TO9) showing C:S and N:S slightly below the accepted criteria has been included in the study and is also evaluated with caution (Table 4). A pig, a sheep/goat and a pike (BANF2, BANF3, CHIN4) have been excluded because the %N, %C, %S, C:S and N:S do not fall within the acceptable range (Table 4).

**Table 4:** δ^15^N, δ^13^C and δ ^34^S values of animal bone collagen.

ID	Chronological period	Species/family	Skeletal element	N (%)	δ^15^N_AIR_ (‰)	C (%)	δ ^13^C_V-PDB_ (‰)	S (%)	δ ^34^S_V-CDT_ (‰)	C:N	C:S	N:S
BANF1	EBA	*Sus domesticus*	humerus	13.7	5.8	37.7	-21.0	-	-	3.2	-	-
*BANF2*	*EBA*	*Ovis aries/Capra hircus*	*tibia*	*3*.*3*	*5*.*3*	*9*.*5*	*-21*.*2*	*0*.*06*	*1*.*8*	*3*.*4*	*399*.*0*	*117*.*4*
*BANF3*	*EBA*	*Sus domesticus*	*humerus*	*0*.*1*	*-*	*1*.*5*	*-*	*0*.*05*	*2*.*9*	*18*.*6*	*88*.*3*	*4*.*8*
BANF5	EBA	*Ovis aries/Capra hircus*	tibia	14.6	5.3	39.7	-21.0	0.20	4.3	3.2	532.2	168.5
BANF6	EBA	*Bos taurus*	talus	10.3	4.8	28.0	-21.0	0.13	3.5	3.2	565.8	179.5
BANF7	EBA	*Bos taurus*	talus	9.9	4.7	27.3	-21.2	0.12	3.8	3.2	588.4	182.8
BANF8	EBA	*Bos taurus*	talus	14.9	3.9	40.6	-21.1	0.20	5.2	3.2	548.2	173.2
RCV4	MBA	*Bos taurus*	humerus	14.3	5.9	39.2	-21.4	0.20	8.4	3.2	403.3	127.1
RCV10	MBA	*Sus domesticus*	mandible	12.7	8.7	34.9	-20.0	0.18	8.7	3.2	431.1	136.2
TO1	FBA	*Ovis aries*	radius	15.1	6.5	41.1	-21.6	0.27	3.1	3.2	389.9	122.3
TO2	FBA	*Bos taurus*	radius	14.7	6.6	40.1	-21.4	0.25	4.7	3.2	339.0	106.2
TO4	FBA	*Cervus elaphus*	radius	15.2	5.9	41.7	-21.3	0.28	1.8	3.2	373.4	116.5
TO5	FBA	*Capra hircus*	ulna	14.2	3.7	39.1	-21.4	0.31	3.7	3.2	331.1	102.1
TO7	FBA	*Sus domesticus*	humerus	14.9	8.5	41.0	-20.3	0.29	3.5	3.2	298.5	91.7
TO8	FBA	*Sus domesticus*	humerus	15.0	6.4	41.8	-20.4	0.33	4.4	3.3	386.2	121.1
TO9	FBA	*Sus domesticus*	mandible	14.2	6.3	39.6	-21.0	0.35	3.2	3.3	335.2	104.5
TO11	FBA	*Bos taurus*	humerus	14.6	2.7	40.0	-22.0	0.27	0.7	3.2	403.3	127.1
TO13	FBA	*Ovis aries*	tibia	14.2	7.0	39.2	-20.7	0.31	4.2	3.2	431.1	136.2
CHIN1	FBA	*Esox lucius*	vertebra	14.9	10.7	45.3	-21.5	0.60	-1.8	3.5	199.1	56.4
CHIN2	FBA	*Esox lucius*	vertebra	14.5	9.8	44.2	-23.4	0.55	-5.7	3.5	213.0	60.3
CHIN3	FBA	*Esox lucius*	vertebra	15.1	10.4	45.3	-23.8	0.61	-5.2	3.5	198.2	57.0
*CHIN4*	*FBA*	*Esox lucius*	*hyomandibular*	*1*.*5*	*10*.*3*	*7*.*0*	*-24*.*1*	*0*.*21*	*-6*.*7*	*5*.*4*	*89*.*2*	*16*.*5*

Sites: Collombey-Muraz, La Barmaz (BANF); Rances, Champ Vully (RCV); Chindrieux (CHIN); Chens-sur-Léman, Tougues (TO).

The terrestrial animals show δ^15^N values between 2.7‰ to 8.7‰ (5.8‰ ± 1.6‰, n = 16), between -22.7‰ to -20.0‰ for δ^13^C (-21.1‰ ± 0.5‰, n = 16) and between 0.7‰ and 8.7‰ for δ^34^S (4.2‰ ± 2.1‰, n = 15). Two of the pigs, RCV10 and TO7, have high nitrogen values suggesting an important animal protein intake, whereas all the other pigs present typical herbivore values.

A Kruskal-Wallis test does not highlight differences for δ^13^C and δ^15^N between the Early, Middle and Final Bronze Age (*p* = 0.758 and *p* = 0.102, respectively), conversely to δ^34^S which displays significant differences (*p* = 0.027) (Table 5). An Exact Wilcoxon Mann Whitney test between the Early and Final Bronze Age animals does not show any significant differences (*p* = 0.212). An Exact Wilcoxon Mann Whitney test for the Middle Bronze Age animals was not run because only two samples are available. However, it is evident that these individuals have higher sulfur values than all the other animals.

**Table 5:** Statistical parameters for the animal bone collagen.

	δ^15^N_AIR_						δ^13^C_V-PDB_				δ^34^S_V-CDT_			
	species	n	min	max	Mean (1sd)	*p* (Kruskas wallis)	min	max	mean (1sd)	*p* (Kruskas wallis)	min	max	mean (1sd)	*p* (Kruskas wallis)
**Collombey-Muraz, La Barmaz Early Bronze Age**	Pig	1	5.8	5.8	-	0.102	-21.0	-21.0	-	0.758	-	-	-	***0*.*027***
	Sheep/goat	1	5.3	5.3	-		-21.0	-21.0	-		4.3	4.3	-	
	cattle	3	3.9	4.8	4.5 (0.5)		-21.2	-21.0	-21.1 (0.1)		3.5	5.2	4.2 (0.9)	
	*TOT*	*5*	*3*.*9*	*5*.*8*	*4*.*9 (0*.*7)*		*-21*.*2*	*-21*.*0*	*-21*.*1 (0*.*1)*		*3*.*5*	*5*.*2*	*4*.*2 (0*.*8)*	
**Rances, Champ Vully Middle Bronze Age**	pig	1	8.7	8.7	-		-20.0	-20.0	-		8.7	8.7	-	
	cattle	1	5.9	5.9	-		-21.4	-21.4	-		8.4	8.4	-	
	*TOT*	*2*	*5*.*9*	*8*.*7*	*7*.*3 (2*.*0)*		*-21*.*4*	*-20*.*0*	*-20*.*7 (1*.*0)*		*8*.*4*	*8*.*7*	*8*.*6 (0*.*2)*	
**Chens sur Léman Final Bronze Age**	pig	3	6.3	8.5	7.1 (1.3)		-21.0	-20.3	-20.6 (0.4)		3.2	4.4	3.7 (0.6)	
	Sheep/goat	3	3.7	7.0	5.7 (1.8)		-21.6	-20.7	-21.2 (0.4)		3.1	4.2	3.7 (0.6)	
	cattle	2	2.7	6.7	4.6 (2.8)		-22.1	-21.4	-21.7 (0.5)		0.7	4.8	2.7 (2.9)	
	Red deer	1	5.9	5.9	-		-21.3	-21.3	-		1.8	1.8	-	
	*TOT*	*9*	*2*.*7*	*8*.*5*	*5*.*9 (1*.*7)*		*-22*.*1*	*-20*.*3*	*-21*.*1 (0*.*4)*		*0*.*7*	*4*.*8*	*3*.*3 (1*.*3)*	
	pike	3	9.8	10.7	10.3 (0.5)		-23.8	-21.5	-22.9 (1.2)		-5.7	-1.8	-4.3 (2.1)	

The three pike range from 9.8‰ to 10.7‰ for δ^15^N (10.3‰ ± 0.5‰, n = 3), between -23.8‰ and -21.5‰ for δ^13^C (-22.9‰ ± 1.2‰, n = 3) and between -5.7‰ and -1.8‰ for δ^34^S (-4.3‰ ± 2.1‰, n = 3). The negative sulfur values are not surprising considering that the δ^34^S of sulfates from riverine ecosystems mainly range from -5 to +15‰, with outliers resulting from geochemical processes of sulfate reduction or sulfide oxidation [61, 116, 117].

Bone collagen was extracted from 41 humans. All the results obtained from La Barmaz and Vufflens respect the quality criteria; three samples from Tolochenaz, LaC1, LaC4 and LaC10, were excluded because the %N and %C values do not respect the accepted ranges. Due to dating problems, LeB1 is excluded from the interpretations.

The δ^15^N values range between 7.2‰ and 10.1‰ (8.6‰ ± 0.8‰, n = 37), δ^13^C between -21.2‰ and -17.6‰ (-19.8‰ ± 1.2‰, n = 37) and δ^34^S between -2.6‰ and 8.9‰ (2.9‰ ± 2.1‰, n = 36) (Table 6).

**Table 6:** δ^15^N and δ^13^C_col_ of human bone and dentine collagen and δ^13^C_enamel_.

						COLLAGEN (bone)									COLLAGEN (dentine)				APATITE (enamel)			
ID	Burial	Skeletal element	Tooth	Sex	Age	N (%)	δ^15^N (‰)	C (%)	δ ^13^C_coll_ (‰)	C/N	%S (%)	δ^34^S (‰)	C/S	N/S	N (%)	δ^15^N (‰)	C (%)	δ^13^C (‰)	Carbonate content	δ^13^C_enamel_ (‰)	Sample weight used (mg)	CO2 (ml)
BA3	N3	ulna R	M_2_ R	F	A	14.1	8.3	38.5	-20.5	3.2	0.21	3.5	485.2	153.0	15.9	9.1	42.8	-20.1	4.16	-14.1	6.07	0.056
BA5	N5	ulna L	M_2_ R	M	A	15.1	8.4	40.9	-20.4	3.2	0.19	-2.6	570.4	181.1	16.4	8.6	45.2	-20.4	2.73	-15.1	5.91	0.036
BA6a/b	N6a/N6b	ulna L	M_2_ R	F	A	12.8	7.8	35.9	-20.1	3.3	0.19	2.0	488.5	150.3	16.9	8.6	45.4	-20.1	3.59	-13.8	5.71	0.046
BA6	N6	ulna L	M_2_ R	F	A	14.9	8.4	40.4	-20.4	3.2	0.18	1.8	580.6	183.9	15.0	8.9	42.1	-20.7	3.16	-14.3	5.95	0.042
BA22	N22	ulna L	M_2_ R	M	A	15.1	8.8	44.6	-20.8	3.4	0.23	0.8	510.0	148.6	16.1	9.6	43.2	-20.8	2.50	-15.4	5.32	0.030
BA23	N23	tibia R	M_2_ R	M	A	16.0	10.1	47.2	-20.5	3.4	0.23	1.6	554.8	161.5	16.0	11.8	43.5	-20.4	3.04	-14.5	5.91	0.040
BA25	N25	ulna L	M_2_ R	M	A	15.1	8.9	45.2	-20.8	3.5	0.26	1.0	470.2	135.4	16.0	8.8	43.5	-20.8	2.54	-14.3	6.10	0.035
BA26	N26	ulna L	M_2_ R	M	A	15.4	10.0	46.1	-20.5	3.5	0.24	-0.8	514.6	148.2	16.6	10.0	44.7	-20.3	3.01	-13.7	6.04	0.041
BA28	N28	humerus R	M^2^ R	F	A	15.9	8.0	47.7	-20.9	3.5	0.26	2.3	484.8	139.4	17.0	7.9	45.7	-21.1	2.87	-15.0	4.44	0.029
BA42	N42	ulna L	M_2_ R	M	YA	16.7	7.7	49.7	-21.2	3.5	0.25	0.8	526.2	151.7	16.6	8.5	45.7	-21.1	3.29	-14.1	4.89	0.036
BA50	T50	ulna L	M_2_ R	F	A	14.0	9.0	41.8	-20.2	3.5	0.22	4.2	493.7	142.6	16.2	9.8	46.5	-20.0	2.70	-12.9	6.17	0.037
BA53	N53	tibia R	-	M	A	15.5	8.2	46.6	-20.9	3.5	0.26	0.8	475.1	136.2	-	-	-	-	-	-	-	-
ZHB	ZHB	ulna L	M_2_ R	M	A	13.1	8.3	37.0	-20.6	3.3	0.19	4.1	505.6	154.6	16.2	8.1	44.5	-21.1	2.98	-14.8	6.02	0.040
VF1	VF 94/st 1 ind 1	ulna R	M_2_ R	F	A	14.9	9.0	44.5	-20.4	3.5	0.25	3.7	479.4	137.9	16.5	8.8	44.6	-20.8	1.47	-13.7	6.15	0.020
VF2	VF 94/st 1 ind 2	fibula	M_2_ R	M	YA	13.8	9.6	41.8	-20.4	3.5	0.22	3.1	505.1	143.3	16.5	8.8	44.3	-20.6	2.29	-13.7	6.07	0.031
VF3	VF 95/st 4 ind.1	humerus R	M_2_ L	F	A	14.5	9.5	43.3	-20.3	3.5	0.28	4.4	410.1	118.4	16.6	10.1	44.7	-20.6	2.82	-13.7	5.25	0.033
VF4	VF 95/st 4 ind 2	humerus L	M_2_ R	I	C	14.6	8.8	43.8	-20.3	3.5	0.25	3.8	457.6	131.0	12.3	9.5	34.1	-20.5	2.77	-13.1	6.04	0.037
VF5	VF 95/st 4 ind 3	ulna R	M^2^ L	F	YA	14.2	10.0	42.9	-20.9	3.5	0.24	2.0	477.0	135.6	14.4	10.3	41.1	-21.5	2.63	-13.7	6.14	0.036
VF6	VF 95/st 4 ind 4	ulna L	C sup R	I	C	13.4	9.5	42.1	-20.9	3.7	0.25	3.6	445.6	122.2	16.0	10.2	43.4	-20.5	3.33	-13.0	6.01	0.045
VF7	VF 95/st 4 ind 5	ulna R	M_2_ R	F	a	14.3	8.6	43.0	-20.5	3.5	0.26	2.1	442.9	126.6	15.3	8.7	43.0	-21.0	2.30	-13.4	5.24	0.027
VF8	VF 95/st 4 ind 6	ulna L	M_2_ L	F	A	14.2	8.7	42.9	-20.5	3.5	0.27	3.7	422.2	120.3	16.1	8.8	44.0	-20.7	2.23	-13.6	5.69	0.028
VF9	VF 95/st 9	radius L	M_2_ L	M	A	13.0	9.8	39.7	-20.5	3.5	0.27	2.6	393.6	111.3	12.8	10.4	35.9	-20.5	3.36	-12.8	5.52	0.042
VF10	VF 95/st 10	radius	M_2_ L	I	C	14.7	8.8	44.4	-20.6	3.5	0.22	2.8	533.0	152.1	16.2	9.3	43.5	-20.8	1.75	-13.6	5.97	0.023
VF11	VF 95/st 11	radius L	M_2_ R	M	a	14.8	8.9	44.5	-20.4	3.5	0.24	2.9	483.1	138.1	15.6	9.7	42.7	-20.4	2.27	-13.9	6.08	0.031
VF12	VF 95/st 14	ulna R	M_2_ L	M	YA	14.7	8.4	44.0	-20.5	3.5	0.25	2.8	474.4	136.2	16.1	8.5	44.4	-21.2	3.38	-13.8	5.34	0.040
LaC1	st. 1052	radius R	M_2_ L	M	A	10.2	8.6	35.8	-17.6	4.1	0.13	7.4	722.9	176.3	16.1	9.6	43.7	-20.3	2.39	-13.3	5.99	0.032
LaC2	st. 1061	radius R	M_2_ R	F	A	14.8	7.3	44.5	-18.0	3.5	0.19	2.8	609.0	174.6	16.1	7.3	43.6	-18.3	2.57	-10.8	5.93	0.034
LaC3	st. 1074	tibia R	M^1^ L	I	A	10.8	8.6	30.5	-18.5	3.3	-	-	-	-	15.5	8.5	42.1	-16.7	2.47	-9.3	5.59	0.031
LaC4	st. 1111	ulna L	PM^1^ L	M (?)	A	6.4	8.6	18.2	-18.2	3.3	0.12	6.7	410.8	124.2	16.4	8.4	44.1	-17.1	2.21	-9.1	6.08	0.030
LaC5	st. 1057	ulna R	M^2^ R	I	C	12.9	7.5	34.8	-18.4	3.2	0.17	5.7	530.3	168.9	16.1	8.0	44.1	-18.3	4.15	-10.6	5.18	0.048
LaC6	st. 1080	humerus	I_2_ R	I	C	11.4	7.7	31.1	-18.4	3.2	0.16	4.6	515.1	162.3	14.9	9.4	40.8	-17.8	3.22	-10.4	5.93	0.043
LaC7	st. 1018	clavicle R	I_2_ R	I	C	14.5	8.1	39.0	-18.3	3.1	0.19	4.0	542.4	173.4	15.1	9.9	40.7	-16.4	2.99	-9.4	5.61	0.038
LaC8	st. 1070	femur L	*-*	I	C	13.8	8.0	37.8	-19.1	3.2	0.18	5.0	545.6	170.8	-	-	-		-		-	-
LaC9	st. 1083	tibia R	*-*	I	A	13.0	7.6	35.2	-17.6	3.2	0.16	4.1	594.1	188.2	-	-	-		-		-	-
LaC10	st. 1071	femur R	*-*	I	A	1.0	-	3.9	-	-	-	-	-	-	-	-	-		-		-	-
*LeB1*	*tb XXIV*	*femur L*	*M*_*2*_ *L*			*14*.*6*	*9*.*2*	*44*.*1*	*-20*.*5*	*3*.*5*	*0*.*23*	*0*.*9*	*514*.*5*	*146*.*9*	*15*.*4*	*9*.*7*	*41*.*6*	*-20*.*3*	*1*.*46*	*-14*.*4*	*6*.*03*	*0*.*020*
LeB2	tb IX-X	femur R	*-*	M		10.4	8.3	31.8	-17.2	3.5	0.20	1.3	423.8	120.0	-	-	-		-		-	-
LeB3		femur R	*-*			15.6	9.7	46.7	-20.4	3.5	0.20	2.9	604.4	173.7	-	-	-		-		-	-
LeB4	tb I	femur R	M_2_ R			13.8	7.2	42.1	-18.2	3.6	0.22	1.2	505.7	142.6	13.8	7.5	38.9	-17.7	3.33	-10.1	5.42	0.040
LeB5	tb III	femur L	M_2_ L			14.1	8.4	42.3	-17.2	3.5	0.20	8.9	556.4	159.8	14.9	10.1	41.2	-17.1	2.70	-9.9	5.92	0.036
LeB6		humerus L	M_2_ R			15.6	8.6	47.0	-17.5	3.5	0.19	6.7	638.9	182.5	15.6	8.5	42.1	-17.4	1.53	-10.3	5.95	0.020

When pooling together all the individuals from the three sites, significant differences appear for δ^15^N (*p* < 0.001), δ^13^C (*p* < 0.001) and δ ^34^S (*p* = 0.002) (Table 7). An Exact Wilcoxon test between pairs of sites (Table B in S1 Text) shows significant differences for δ^13^C values for Tolochenaz when compared to Vufflens (*p* < 0.001) and La Barmaz (*p* < 0.001). For δ^15^N, slight differences are evident between La Barmaz and Vufflens (*p* = 0.040) and between Vufflens and Tolochenaz (*p* < 0.001). For δ^34^S, differences are present between La Barmaz and Vufflens (*p* = 0.009) and between La Barmaz and Tolochenaz (*p* = 0.004). Differences within each community have also been tested according to biological parameters. Given the number of individuals, statistical analyses were performed only on La Barmaz and Vufflens when considering sex differences. No statistically significant differences appear within these communities (Table C in S1 Text). Concerning age-at-death, statistical analysis was only possible for Vufflens and no statistically significant differences have been detected (Table C in S1 Text). When considering presence-absence of grave goods, no differences are highlighted for La Barmaz (δ^13^C, *p* = 0.508; δ^15^N, *p* = 0.086; δ^34^S, *p* = 0.422). For Vufflens and Tolochenaz, given the difficulties in attributing specific grave goods to some individuals, statistical analyses were not performed for this parameter.

**Table 7:** Statistical parameters of isotope data for bone and dentine human collagen and apatite.

	δ^15^N				δ^13^C_coll_				δ^34^S				δ^13^C_enamel_			
**Collombey-Muraz, La Barmaz**	**Bone**															
	n	**min**	**max**	**Mean (1sd)**	n	**min**	**max**	**Mean (1sd)**	n	**min**	**max**	**Mean (1sd)**				
	13	7.7	10.1	8.6 (0.7)	13	-21.2	-20.1	-20.6 (0.3)	13	-0.8	4.2	1.9 (1.4)				
	**Dentine**												**Enamel**			
	n	**min**	**max**	**Mean (1sd)**	n	**min**	**max**	**Mean (1sd)**					n	**min**	**max**	**Mean (1sd)**
	12	7.9	11.8	9.2 (1.1)	12	-21.1	-20.0	-20.6 (0.4)					12	-15.4	-12.9	-14.3 (0.7)
**Vufflens-la-Ville, En Sency**	**Bone**															
	n	**min**	**max**	**Mean (1sd)**	n	**min**	**max**	**Mean (1sd)**	n	**min**	**max**	**Mean (1sd)**				
	12	8.4	10.0	9.1 (0.5)	12	-20.9	-20.3	-20.5 (0.2)	12	2.0	4.4	3.1 (0.7)				
	**Dentine**												**Enamel**			
	n	**min**	**max**	**Mean (1sd)**	n	**min**	**max**	**Mean (1sd)**					n	**min**	**max**	**Mean (1sd)**
	12	8.5	10.4	9.4 (0.7)	12	-21.5	-20.4	-20.8 (0.3)					12	-13.7	-12.8	-13.5 (0.3)
**Tolochenaz, Le Boiron, La Caroline**	**Bone**															
	n	**min**	**max**	**Mean (1sd)**	n	**min**	**max**	**Mean (1sd)**	n	**min**	**max**	**Mean (1sd)**				
	12	7.3	9.7	8.1 (0.7)	12	-20.4	-17.2	-18.2 (0.9)	11	1.2	8.9	4.3 (2.3)				
	**Dentine**												**Enamel**			
	n	**min**	**max**	**Mean (1sd)**	n	**min**	**max**	**Mean (1sd)**					n	**min**	**max**	**Mean (1sd)**
	10	7.3	10.1	8.7 (1.1)	10	-20.3	-16.4	-17.5 (0.7)					10	-13.3	-9.1	-10.3 (1.2)
***p* kruskal Wallis (bone)**	n 37	***p* < 0.001**			n 37	***p* < 0.001**			n 36	***p* = 0.002**						
***P* kruskal Wallis (dentine)**	n 34	*p* = 0.124			n 34	***p* < 0.001**							***p* kruskal Wallis (enamel)**	**N 34**	***p* < 0.001**	

Dentine collagen was extracted from 35 individuals and all the samples respect the quality conditions. The δ^15^N values range between 7.3‰ and 10.4‰ (9.1‰ ± 0.9‰, n = 34), for δ^13^C between -21.5‰ and -16.4‰ (-19.8‰ ± 1.5‰, n = 34). Significant differences occur for δ^13^C (*p* < 0.001) (Table 7). An Exact Wilcoxon test highlights differences for δ^13^C between La Barmaz and Tolochenaz (*p* < 0.001) and between Vufflens and Tolochenaz (*p* < 0.001) (Table B in S1 Text). Like for the bone collagen results, Tolochenaz δ^13^C values are more enriched in ^13^C compared to the other two sites. The test highlights no differences in δ^15^N values among the three communities. In terms of biological criteria, differences by sex are non-significant. With regards to age-at-death, statistical analysis is only possible for Vufflens and no differences are detected (Table C in S1 Text). No differences are present in La Barmaz when results are analyzed by presence-absence of grave goods (δ^13^C, *p* = 0.158; δ^15^N, *p* = 0.222).

The presence of significant differences between the bone and the teeth values of each individual within each human group were tested. The Exact Wilcoxon test for dependent samples highlights differences in La Barmaz for the δ^15^N values, as teeth values are higher than those from bone (*p* = 0.024). In Vufflens and Tolochenaz differences are evident for δ^13^C values. In the Vufflens individuals, bone has higher δ^13^C values than teeth (*p* = 0.019), whereas in Tolochenaz the opposite trend is observable, teeth being enriched in ^13^C as opposed to bone (*p* = 0.046) (Table 8).

**Table 8:** Exact Wilcoxon-Pratt signed-rank test for bone and dentine collagen.

Collombey-Muraz, La Barmaz	Vufflens-la-Ville, En Sency	Tolochenaz, Le Boiron, La Caroline
δ^13^C_,_ *p* = 0.835	δ^13^C_,_ *p* = **0.019**	δ^13^C_,_ *p* = **0.046**
δ^15^N_,_ *p* = **0.024**	δ^15^N_,_ *p* = 0.066	δ^15^N_,_ *p* = 0.093

For the tooth enamel carbonates, 34 individuals were analyzed for δ^13^C_enamel._ Values range from -15.4‰ to -9.1‰ (-12.9 ± 1.5‰, n = 34). Significant differences are present among the three sites (Exact Kruskal Wallis test, *p* < 0.001) (Tables 6 and 7, S4 Fig). A Wilcoxon test comparing the sites by pairs reveals significant differences (Table B in S1 Text). The three groups clearly show different patterns indicating specific diets. Like for the bone and the dentine collagen, Tolochenaz displays higher δ^13^C values compared to the other two groups. Vufflens compared to La Barmaz also shows slightly higher δ^13^C values than La Barmaz. No significant differences are observable between La Barmaz and Vufflens according to sex and age-at-death (Table C in S1 Text).

## Discussion

### Soil management and farming strategies

**Fig 3:**
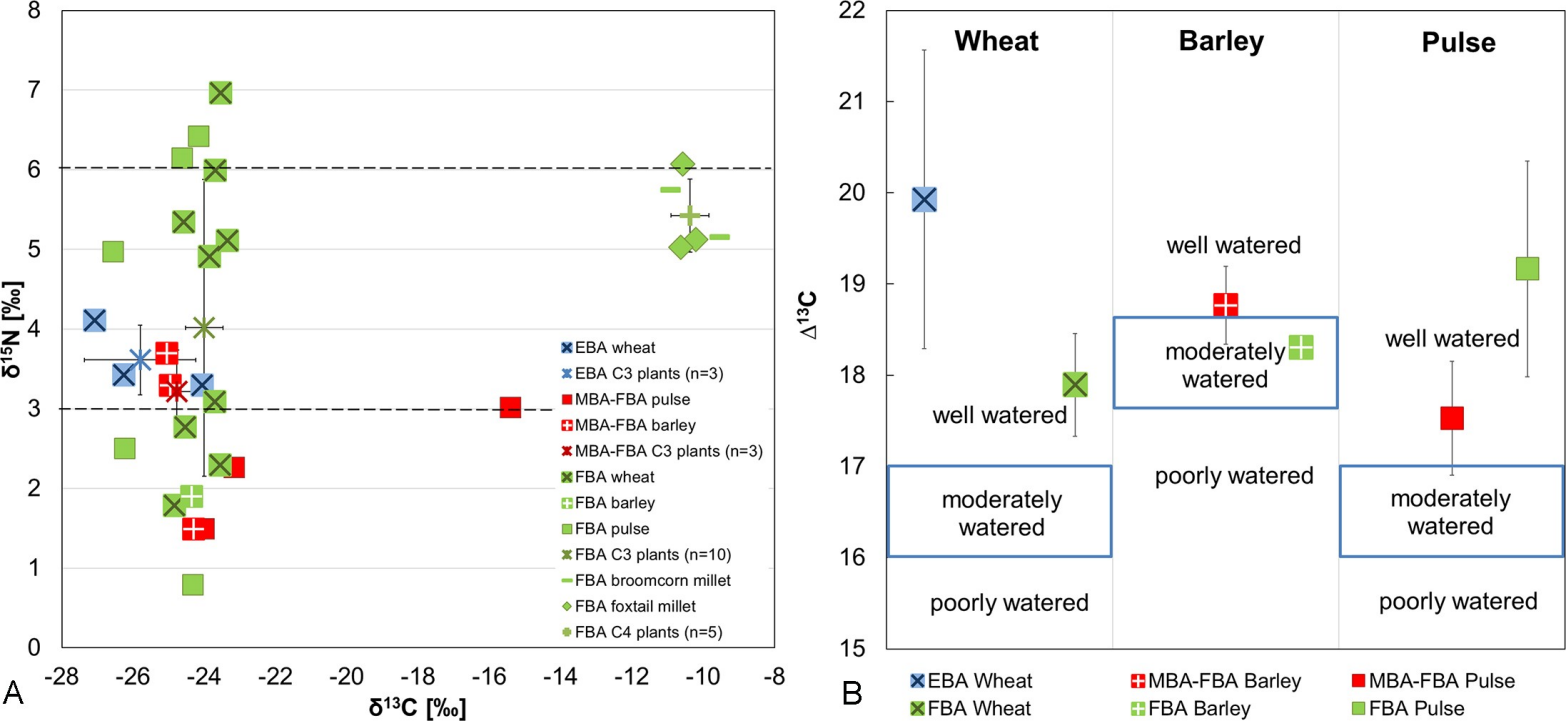
Stable carbon and nitrogen isotope ratios for the archaeobotanical remains. Corrected values have been used for a) δ^15^N results (-0.31‰ for the grains and +2.4‰ for the chaff to eliminate the charring effect) and b) δ^13^C results (-0.11‰ for the grains and +1.9‰ for the chaff). A) Individual data and means (1SD) values are reported for C_3_ plants (wheat and barley) and C_4_ plants (foxtail and broomcorn millet) according to the chronological phases; B) Water status measured according to the ranges proposed by [104].

The wide δ^15^N plant range (Δ^15^N = 5.9) reveals an important variability in nitrogen isotopic values. The use of manure to improve soil fertility leads to an enrichment in ^15^N of cultivated plants, proportional to the duration and intensity of fertilization. According to the δ^15^N ranges established by [70], even though a limited number of samples are present for the Early and Middle Bronze Age, the present data show a certain degree of homogeneity in δ^15^N values for these two periods because most of them are between 3 and 4‰. Conversely, the results of the final phases of the Bronze Age show 1) a greater variability and 2) an enrichment in ^15^N compared to the previous chronological phases (Fig 3A). This could be due to an increase in the use of manure during the Final Bronze Age. Moreover, all the samples come from the same site which means the change in the isotopic signal can be followed over time, a change that could mainly reflect modifications in human agricultural practices because the settlement had a continuous chronology. Millets and legumes warrant particular attention. Not only are millets well represented in this and other coeval sites [44, 46, 118–121], but all the foxtail and broomcorn millet seeds of this settlement show δ^15^N values higher than 5‰. These figures could suggest that these plants were well manured, supporting the importance of these crops in the local economy and, presumably, in the human and/or animal diet. Legumes, whose values are usually lower than those of cereal plants, are less sensitive to the effects of manuring. According to [70], only extremely intensive manuring (> 35 ton/ha for a prolonged duration) increases legume δ^15^N considerably above 0‰. Here, pulses, mainly represented by beans, show values of up to 6.4‰, indicating a high level of fertilization. Such results seem to point to intensified agricultural activity and the increased impact of human actions on farming produce, supporting the idea of more intense agricultural practices during the Bronze Age. This result is in line with the increased demographic pressure suggested by the growing number of settlements and necropoles around Lake Geneva during that time.

**Fig 4:**
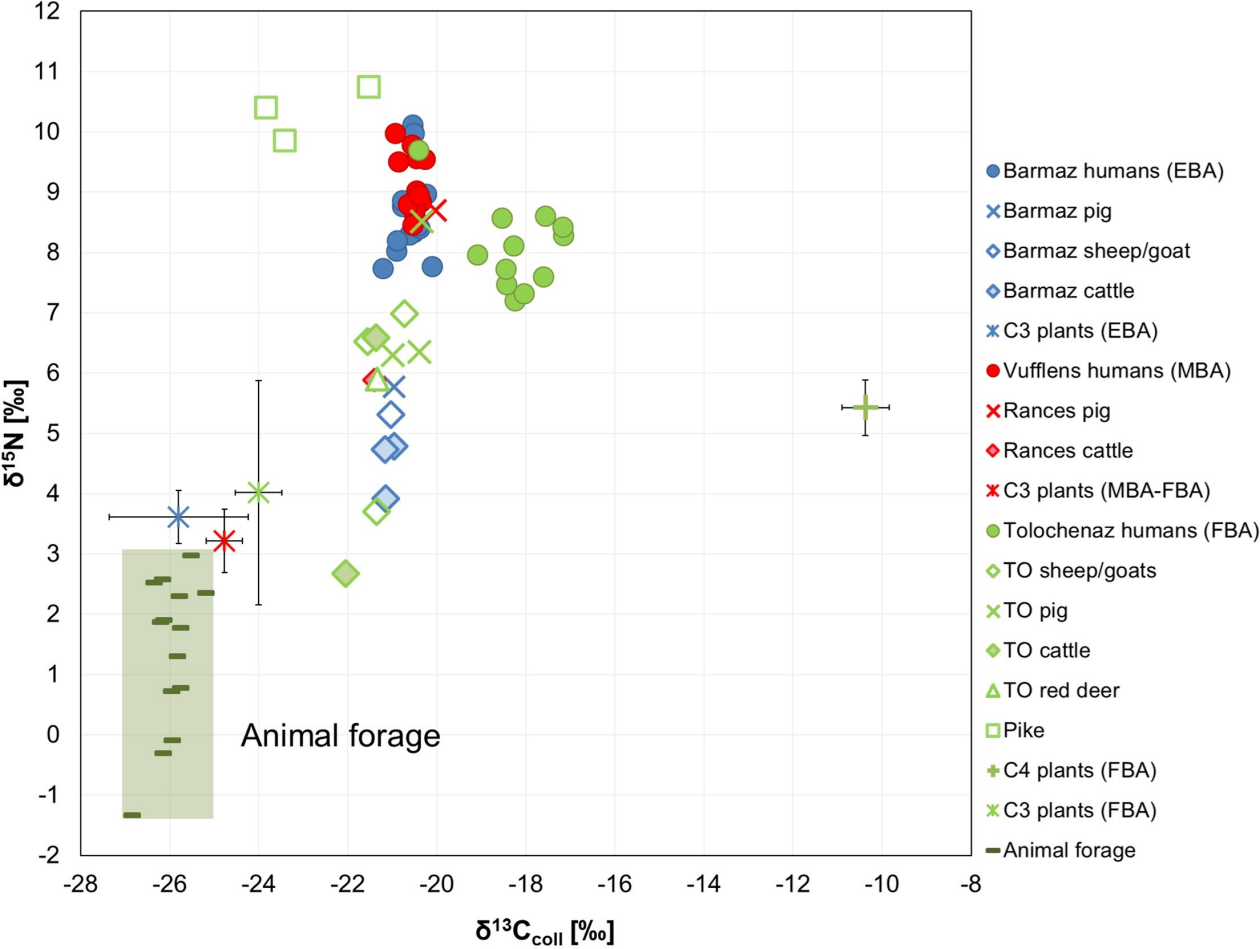
Scatter plot of δ^13^C and δ^15^N bone collagen values for human, animal and botanical remains according to their chronological phases. C_3_ plants include wheat and barley, C_4_ plants include broomcorn and foxtail millets (representation of mean and 1SD for the plants). Abbreviations: TO = Chens sur Léman, Tougues.

An additional parameter to define agricultural and pastoral strategies is the δ^15^N values from wild and domesticated herbivores. From these animal data, it is possible to estimate the values of the plants used to feed them. By inferring the herbivore forage isotope values from bone collagen it is possible to assess the stable isotope values of crops against those of forage. The estimated herbivore forage values represent a “sampling” of local vegetation to which crop values can be compared with to infer agricultural strategies and human practices like manuring and irrigation [69, 75]. The δ^15^N values of the estimated forage suggest that the plants consumed by these animals do not fit within the intervals of the fertilized crops (Fig 4). Two hypotheses can therefore be proposed: the first is that the animals mainly consumed wild plants, likely from more humid or forested areas, while the second presupposes a separate cultivation of plants for fodder. If the latter proposition is retained, it is possible that these crops either underwent less intense fertilization compared to crops for human consumption or that they came from unmanured pastures.

Carbon values of botanical remains reflect the water conditions from which plants have grown [71, 104]. Taking into account the CO_2_ changes that occurred throughout the Holocene, the plant water availability was estimated using the Δ^13^C values (online resource: http://web.udl.es/usuaris/x3845331/AIRCO2_LOESS.xls; [71]), making it possible to compare archaeological data over time. Although the quality and quantity is different according to each Bronze Age phase, the results highlight that barley, wheat and legumes did not suffer hydric stress (Fig 3B). Paths lined by small ditches and small artificial channel structures that canalized water towards fields were discovered in the site from which the carpological remains come from [47], suggesting that water was provided by humans through irrigation infrastructures. Usually, Δ^13^C values for barley grains are ≈ 1‰ higher than those for wheat grown in similar water conditions, mainly because of a difference in crop cycles [104]. In this study, barley shows lower Δ^13^C values compared to wheat and pulses. It is, therefore, likely that barley was less watered than the other species. If the main crops were more tended to through irrigation, these results suggest that wheat and pulses were more important for cultivation than barley. In contrast to the archaeobotanical analysis of the site which indicates that barley was the main crop [122], this suggestion is, nevertheless, consistent with other local and western Switzerland results where wheat prevails [42, 119–121].

Despite the small archaeobotanical sample size making it difficult to present a global scenario for potential changes in farming practices over time, these results are comparable to those of other diachronic Bronze Age studies in southern Europe, where a similar surge in manuring practices and general good water conditions have also been observed [123].

### Reconstructing dietary behaviors

#### Domestic and wild animal feedings

The δ^15^N and δ^13^C results for domestic herbivores (sheep/goat, cattle and pig) show no significant differences for carbon and nitrogen values. This reveals the global homogeneity of isotopic values throughout the Bronze Age (Fig 4). Notwithstanding, statistical differences occur for sulfur values (Table 5): the Rances animals have higher δ^34^S values than those of La Barmaz and Chens-sur-Léman (δ^34^S mean = 8.6‰ vs 4.2‰ and 3.3‰, respectively*)*. Rances is situated further north than the other sites. It is possible that the environment where these animals lived was slightly different from the others, affecting the local values at the base of the food chain and not necessarily implying a diverse diet. All the animals, in fact, indicate a consumption of resources from a temperate environment, mostly constituted of C_3_ type plants. As previously mentioned, the fodder for the domestic herbivores does not appear to have been manured, indicating that these animals could have consumed wild plants, or non-fertilized cultivated plants (Fig 4). Regardless, given that the animal results are all homogeneous, no differential livestock strategies are detected for the Bronze Age.

The three pike values, analyzed in order to understand the possible contribution of aquatic resources to the human diet, are in line with archaeological freshwater fish values in an Alpine environment [124].

#### The human diet

Throughout the sites and phases the local resources are similar, the three human groups can be directly compared. Any differences detected in the human diets must, therefore, depend on different dietary choices. Significant differences for δ^13^C, δ^15^N and δ^34^S among the three groups are present, suggesting changes in dietary habits throughout the Bronze Age (Fig 4). These results are likely due to 1) a greater variability in food consumption over time and 2) the introduction of new resources in the diet at the end of the Bronze Age. The wide distribution of δ^13^C values indicates diverse consumption of C_3_ and C_4_ plants, whereas the δ^15^N and δ^34^S ranges highlight different animal protein intake and, most likely, from different areas.

**Fig 5:**
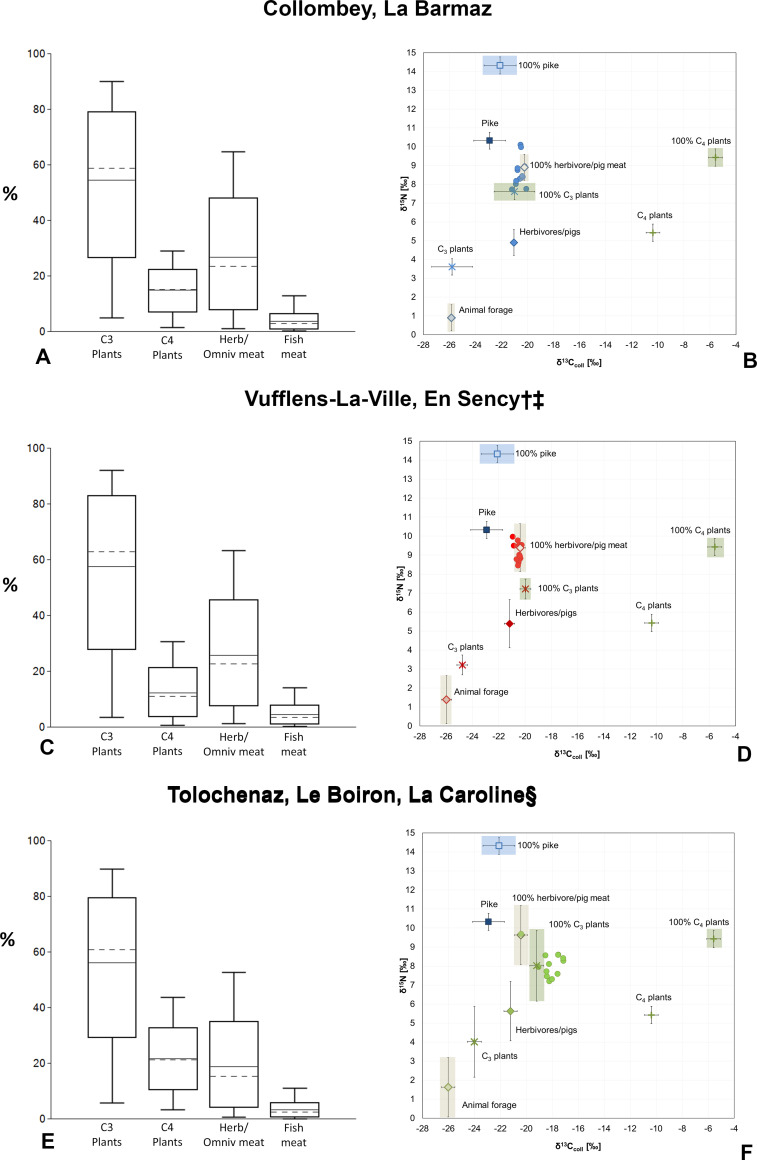
The graphs a), c) and e) represent the contributions of foodstuffs as estimated by the Bayesian model FRUITS using δ^13^C and δ^15^N from human and animal bone collagen and C_3_ and C_4_ cereal plants (for the values entered refer to S1 Table). The graphs b), d) and f) illustrate the individual human bone collagen values and the mean isotope values of the main components of the available foodweb resources. C_3_ and C_4_ plant, freshwater fish and herbivore/pig values (with a herbivore diet) represent the means (±1SD) measured for each Bronze Age phase. Animal forage is evaluated by subtracting the isotopic offsets between plants and consumer collagen (-4.8‰ and -4‰ for the δ^13^C and δ^15^N animal means, respectively); 100% C_3_ plants, 100% C_4_ plants, 100% herbivore/pig meat and 100% pike are estimates of the mean isotopic composition of collagen values for consumers that are one trophic level above the respective resources (+4‰ for δ^15^N and +0.8‰ for δ^13^C for collagen and +4‰ for δ^15^N and +4.8‰ for δ^13^C for plants consumers). ^†^ MBA-FBA cereals. ^‡^ The animals which showed a herbivore diet are considered together for the three BA phases due to the limited number of individuals for the MBA (see the main text for further details). ^§^ FBA animals with a herbivore diet.

The bone collagen δ^15^N and δ^13^C values of the Early Bronze Age La Barmaz humans suggest a mixed terrestrial diet mainly based on C_3_ plants and animal resources (Fig 4). This is supported by the offset between humans and animals, Δ^13^C_humans-animals_ = 0.5‰ and Δ^15^N_humans-animals_ = 3.7‰ (Table D in S1 Text) which are typical values falling within the ranges estimated between two consecutive trophic level. Furthermore, the quite narrow Δ^13^C and Δ^15^N spacings (Δ^13^C = 1.1‰ and Δ^15^N = 2.3‰) suggest that these individuals consumed similar foodstuffs. By applying a Bayesian model like FRUITS which takes into account the coeval isotopic data for animal and plant remains, the contribution of the different food sources to the diet can be better estimated. Fig 5A shows the scenario elaborated by FRUITS (S1 Table). Cereals like wheat and barley played a major role in the diet, representing 54.5%. They are followed by animal products, whose contribution is estimated at almost 30% (26.8%). The importance of animal foodstuff is also supported by the Δ^13^C_humans-plants_ and Δ^15^N_humans-plants_ offsets, which equal 5.2‰ and 5‰, respectively, and are higher than expected from just C_3_ plant consumption alone (Table D in S1 Text). This hypothesis is also confirmed by the scenario presented in Fig 5B, where the La Barmaz humans fit between the ranges of 100% C_3_ plant diet and 100% herbivore and pig meat diet. In addition, legumes may have also contributed to the protein intake but as no data are available for these plants it is not possible to estimate their contribution. Little C_4_ plants (14.9%) seem to have been consumed by this community.

Sulfur results support an intake of terrestrial resources. All the δ^34^S values are below 6‰ and most of them are between 0‰ and 6‰, the typical range for the terrestrial environment. Furthermore, no significant correlation is observed between δ^15^N and δ^34^S values (Rho = -0.11, *p* = 0.70). Two individuals, BA5 and BA26, have negative δ^34^S values. Considering that all the pikes have negative values, freshwater fish cannot be excluded from the diet of these two people. Moreover, δ^15^N values are also quite enriched in ^15^N (BA5 = 8.4‰ and BA26 = 10.0‰). However, a significant consumption of freshwater fish is less likely for the rest of the group as the δ^13^C and δ^34^S values reflect a more terrestrial diet and, according to the FRUITS’ model, the contribution of fish to the diet is very low (3.7%).

When considering the anthropological data, no dietary differences are recorded according to age and sex (Table C in S1 Text). Indeed, this suggests that sex and age probably did not lead to differential dietary practices. A similar assessment is proposed in northern Italy [22, 125] and in central France [126], suggesting an intra-group homogeneity in dietary patterns for the Early Bronze Age. However, some exceptions have been recorded, like in southern Germany [23] and in Poland, where small differences in favor of elder men suggest that mature adults had a diet more enriched in animal protein [52, 127].

The δ^15^N and δ^13^C bone collagen values from the Middle Bronze Age individuals of Vufflens indicate a mixed terrestrial diet, which would have been similar to those from La Barmaz. In order to estimate the Δ^13^C and Δ^15^N offsets between humans and animals of the Middle Bronze Age, the animals from the three phases which displayed a herbivore diet were grouped together. The Δ^13^C_humans-animals_ (= 0.7‰) and Δ^15^N_humans-animals_ (= 3.7‰) values are typical collagen offsets recorded between two adjacent trophic levels. The δ^15^N and δ^13^C human values display a limited range (Δ^13^C = 0.6‰ and Δ^15^N = 1.6‰). The dietary intake was, therefore, rather uniform within this community. According to FRUITS’ model, it seems C_3_ cereal plants were the main component of the diet (Fig 5C), with an estimated contribution equal to 57.6%. The animal protein intake is estimated at 25.6%. The Δ^15^N_humans-plants_ offset is quite important, 5.9‰, suggesting additional resources other than plant based. On the contrary, the Δ^13^C_humans-plants_ offset is 4.3‰ which is slightly lower than the offset estimated between plants and their consumers (4.8‰). This scenario contrasts, to some extent, with the one observed at La Barmaz, where according to FRUITS, the C_3_ plant contribution was lower than in Vufflens (54.5% vs. 57.6%) and animal intake higher (26.8‰ vs. 25.6‰). The differences are quite small and given that these estimates present some uncertainties (S1 Table), it is likely that minor differences in food intake are detected with difficulty. Furthermore, there are some resources like pulses whose contribution is difficult to evaluate. Indeed, pulses values overlap with the C_3_ plants, making their consumption even more difficult to estimate because their relative intake cannot be distinguished. However, the Vufflens individuals are above the range of 100% C_3_ plant diet and within the range of 100% herbivore and pig meat diet (Fig 5D), suggesting a significant role of animal products in the diet of this community. In this case, an eventual consumption of legumes could have been masked by a considerable intake of animal protein. Additionally, given that no significant differences occur between the different Bronze Age animal groups and very few are present between the C_3_ cereal plants of the two phases, the individuals of La Barmaz and Vufflens are directly comparable. Though both the carbon and nitrogen ranges mainly overlap, significant differences are observable for the δ^15^N values (Table B in S1 Text, Fig 4), confirming a difference in terms of animal protein intake between the two communities. As for the C_4_ plants, they were not a main component of the diet in the Vufflens humans, nor was freshwater fish (Fig 5C and 5D). Sulfur data confirm the main C_3_ terrestrial origin of the resources consumed. The δ^34^S mean is 3.1‰ with a limited distribution (Δ^34^S = 2.4‰) and no significant correlation is observed between δ^15^N and δ^34^S (Rho = -0.08, *p* = 0.78). Like in the La Barmaz cemetery, no differences in food habits are recorded that correlate with the anthropological data and the funerary treatment. Moreover, the statistical analyses do not reveal any differences either (Table C in S1 Text).

The Final Bronze Age results from the Tolochenaz humans clearly differ from the two previous communities, particularly the carbon data. First, the δ^13^C, δ^15^N and δ^34^S values of Tolochenaz are more widespread compared to those of La Barmaz and Vufflens, suggesting a more varied diet within this group. Then, the Δ^13^C_humans-animals_ offset of 3‰ (min = –20.4‰ max = -17.2‰), highlights a mixed C_3_-C_4_ diet, with a remarkable enrichment in ^13^C for the human values. This reflects C_4_ plants like millets intake. As for the nitrogen, a Δ^15^N_humans-animals_ offset of 2.5‰ indicates a rather limited consumption of animal proteins (Fig 4). This is confirmed by the FRUITS’ model, where even if the main staple was C_3_ cereal plants (56.1%), C_4_ plants played an important role in the diet (21.6%), in addition to a less important animal product intake (18.8%). The freshwater fish contribution was minimal (3.3%) (Fig 5E). This scenario is confirmed in Fig 5F, where the Tolochenaz humans plot below the 100% herbivore and pig meat range, and are slightly to left of the 100% C_3_ plant range because of the contribution of millets. Sulfur results support the main terrestrial origin of the resources consumed, confirmed by the absence of a significant correlation between δ^15^N and δ^34^S (Rho = 0.36, *p* = 0.33). No differences are evident when analyzing the results according to the individuals’ associated grave goods. Nevertheless, only LeB3, differs from the general pattern. His/her δ^13^C value of -20.4‰ is typical of a diet based on C_3_ resources and the δ^15^N is the highest, 9.7‰, suggesting a greater animal protein intake than for the rest of the group. Indeed, this result fits perfectly in the range of the 100% animal diet (Fig 5F). However, his/her δ^34^S value (2.9‰) is consistent with the other Tolochenaz results. It is therefore not possible to ascertain whether this individual came originally from a different place or not. Moreover, since his/her sex and age were not evaluated because of the bad preservation of the remains and no special grave goods were present, it is difficult to formulate a hypothesis to explain the difference in diet. It is possible he/she came from another community, where different food habits could have been in place. Discussion about mobility is further approached in another paper, where additional strontium and oxygen isotope data are considered.

#### Reconstruction of dietary changes during the Bronze Age

This research shows differences in dietary practices both within and among communities throughout the Bronze Age in a region that is geographically restricted. Whilst during the first phases of the Bronze Age, barley, einkorn and wheat are the basis of the diet, during the Final Bronze Age broomcorn and foxtail millet are introduced, becoming an important component of the diet, as confirmed in the following phases at least for some individuals [60, 128]. This could be due to the introduction of new agricultural practices following new social and economic contacts with southern Europe societies where these crops were already present [20, 21, 25]. Another factor could be the intensification of anthropic activities on the environment due to population growth, among which would be deforestation and diversified land use–forestry, agriculture and pastoralism. These human activities could have strongly impacted soil conditions causing dryness, responsible for the decrease in soil fertility, which in turn could have favored the farming of more resistant crops, better adapted to harsh and extreme conditions, like millets. In addition, the influence of climate fluctuation during the Bronze Age needs to be considered. It could have contributed to the increase of soil aridity and pushed humans to look for and adopt new staple crops [129]. The combination of these events was responsible, on the one hand, for the development of alternative strategies for more efficient and fast agricultural production, and on the other hand, for the diffusion of new species. Even though in some areas, like in northern and central Italy, this trend appears earlier, from the Early-Middle Bronze Age transition [20, 21, 24, 25], these results confirm the general European and western Asian trend. Isotopic studies on humans and animals in France [126, 130], Germany [23, 131, 132], Austria [133], Hungary [134] Slovenia [135], Croatia [136] indicate millet consumption from the Final Bronze Age onwards. Switzerland, with a similar pattern, could be part of this “Euro-Asian wave” of new feeding practices, showcasing how this region is part of and well connected to the rest of Europe. The geomorphology of the area in which the sites are located, a wide Plateau surrounded by the Alps, is not a barrier to the diffusion of new activities and subsistence strategies. On the contrary, the mountains and the Plateau could in fact represent a source of unity because they are rich in raw materials, indispensable to the populations of the foothills, encouraging mobility and exchanges at different levels.

The consumption of animal products also changes over time. During the Early and Middle Bronze Age, meat and derived products seem to be quite significant, whereas during the Final Bronze Age they are less important. Low animal protein consumption at the end of the Bronze Age-beginning of the Iron Age is recorded in other European areas [137]. This decrease could be the result of a defined economic strategy directed to favor crop cultivation as opposed to animal husbandry, as suggested by the intense use of manure as well as the increase in crop diversity during the Final Bronze Age. The reason for these choices could be that population growth is easier to sustain through cultivated foodstuffs instead of animal meat production or the production of secondary animal products.

The three groups do not show any aquatic resource exploitation, despite being close to rivers and lakes. However, the occasional consumption of fish cannot be excluded. Its intake could have been so low that it would not be detected through isotope analysis.

Regarding potential differences in dietary patterns linked to individual social status, ethnological research has demonstrated that high social status privileges include differential access to food, with certain foodstuffs being exclusively set aside for rulers or the ruling lineage [138]. When comparing individuals in Early and Middle Bronze Age cemeteries with different grave goods, almost no differences in terms of diet and geographic origin appear [20, 22, 126], with few exceptions [139]. On the contrary, differences are observable from the end of the Bronze Age [21, 140, 141] and sometimes they seem more linked to the funerary structure than to the grave goods [136]. During the Iron Age, men buried with weapons often have diets with a higher animal protein component than other individuals [59, 132, 133, 142]. Among the communities studied here, high social status individuals have not been identified through the archaeological material except for those from Vufflens, where all the individuals likely belong to the local elite [31]. However, Vufflens is the only Middle Bronze Age community isotopically studied to date in this area. Then, since the Middle Bronze Age data are similar to that of the Early Bronze Age, dietary trends seem consistent throughout the earlier Bronze Age periods and no specific foodstuffs appear to have been consumed by high-status individuals.

#### Juvenile vs. adult dietary patterns

**Fig 6:**
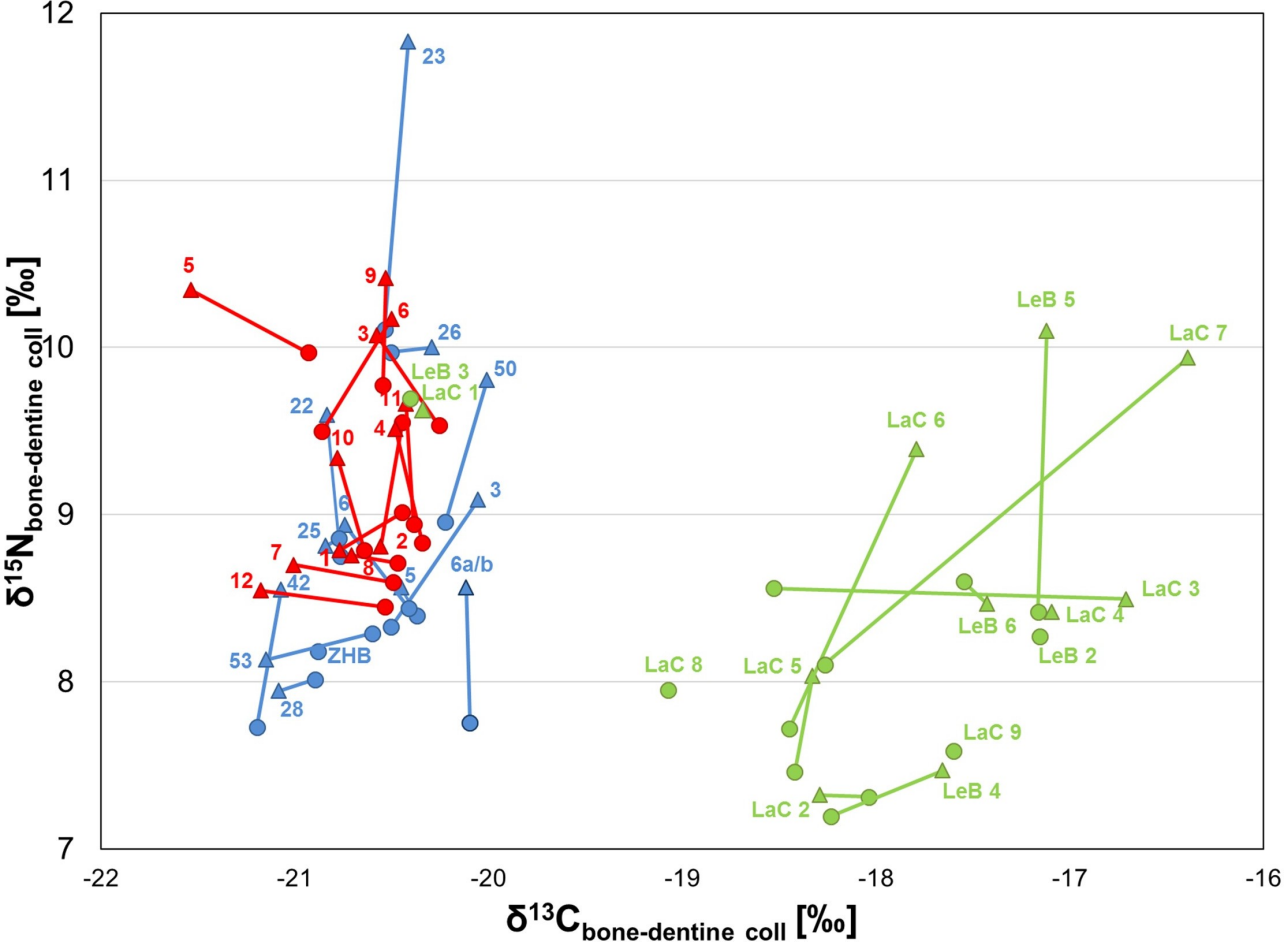
Scatter plot of the δ^13^C and δ^15^N values of dentine (triangle) and bone (circle) collagen at the three sites. Data points are labelled with grave numbers.

The bone and dentine δ^15^N and δ^13^C collagen results indicate that diet did not differ significantly between childhood and adulthood (Fig 6). This means that the general food habits were not age dependent, but rather, were characteristic of each community. In fact, in La Barmaz and Vufflens the data suggest a prevalent C_3_ diet from infancy, whereas in Tolochenaz the contribution of millets is highlighted from infancy, because both dentine and bone show high δ^13^C values (Fig 6). Furthermore, as already observed, the La Barmaz and Vufflens bone collagen δ^15^N and δ^13^C results have a narrow distribution, suggesting consistent dietary choices within each group, contrary to Tolochenaz, whose δ^13^C and δ^15^N values show a wider distribution. These trends can also be observed for collagen dentine results. The Tolochenaz δ^13^C and δ^15^N ranges are larger than the La Barmaz and Vufflens ranges (excluding from the δ^15^N values of La Barmaz the outlier BA23; S5 Fig). Consequently, for the Final Bronze Age, as opposed to the previous periods, both children and adults have access to a wide variety of food resources.

Two general patterns are evident in all sites. First, the dentine values range more widely than the bone ones. Second, the δ^15^N values are higher in dentine than in bone, even if only La Barmaz shows statistical differences (*p* = 0.024) (Fig 6, Table 7). These two trend are in all likelihood linked to a greater consumption of animal protein during childhood. The Δ^15^N_dentine-bone_ individual values of La Barmaz range from -0.2‰ to 1.7‰, which can be explained through a greater meat and/or dairy food intake in childhood compared to adulthood. However, as similar trends have already been highlighted in other communities, other factors, especially physiological ones, may have caused the enrichment in ^15^N in dentine [143, 144]. Changes in δ^15^N values can be also related to extended periods of breastfeeding, episodes of physiological stress and catabolism generated by starvation, long-term disease (infection, injury) or harsh climatic conditions [145–150]. In catabolic states, the organism uses amino acids from its own body tissues to synthesize new proteins, leading to an increase of δ^15^N values [147, 151–153]. Elevated δ^15^N levels could also result from a catabolism related to malnutrition [152, 154]. Enamel hypoplasia has been identified in several individuals (e.g., BA6, BA6a/b), but given its complex etiology, it is possible that malnutrition or other physiological stress resulted in fluctuations in the δ^15^N collagen values of these individuals.

In terms of the Δ^13^C_dentine-bone_ values, statistical differences are present in Vufflens and Tolochenaz (*p* = 0.019 and *p* = 0.046, respectively). The difference between the dentine and bone means are 0.3‰ in Vufflens and 0.6‰ in Tolochenaz. A value like 0.3‰ could just reflect the normal fluctuation over a person’s lifetime, whilst 0.6‰ is quite considerable, since it is almost a step between two consecutive trophic levels. Particularly, LaC3 and LaC7 show wide offsets of 1.8‰ and 1.9‰, respectively. Even if for both individuals, bone and dentine values confirm a substantial millet intake, teeth show higher δ^13^C compared to bone. It seems that millet was more important in childhood than in adulthood. This result is apparent in most individuals. While elevated δ^15^N can represent both nutritional and physiological stress, a metabolic relationship with δ^13^C is limited [155]. Thus, variations in δ^13^C values reflect differential contributions of C_3_ and C_4_ plants. Isotope values do not seem to highlight any differences according to biological criteria such as age and sex. These elements appear therefore to have no impact on dietary choices.

Two individuals from Tolochenaz, LeB3 and LaC1, stand out because their diets differ from the rest of the community (Fig 6). LeB3 has already been discussed for the diet mainly based on C_3_ plants as an adult, but no teeth are available to confirm this trend in childhood as well. Concerning LaC1, both bone and dentine were analyzed. The bone values indicate bad preservation and were therefore not included in the previous discussion. However, if the results are correct, he would be the only individual with a drastic dietary change during his life. During childhood he would have mainly had a C_3_ diet (δ^13^C = -20.3‰), while in adulthood he would have had a mixed C_3_-C_4_ diet (δ^13^C = -17.6‰). He was a mature adult man, with a callus bone in the right ulna resulting from a fracture [35]. Unfortunately, the discussion for this individual can go no further for obvious reasons, but some interesting questions arise. If the results are acceptable, why is he the only one with a dietary shift? Even if a definitive answer is not possible, this study identifies the presence of individuals with different dietary habits, likely linked to mobility events. Indeed, this result may support exchanges between different communities with diverse eating traditions. Investigations on strontium and oxygen isotopes provide further details about this individual’s life [156].

#### The apatite data

Carbon in apatite is a useful proxy to detect the contribution of low trophic level food, representing a supplementary trophic marker [157–159]. It helps determine marine vs. terrestrial diets and C_3_ vs. C_4_ plant intake [80, 86, 157, 160]. Combining collagen and apatite data through the apatite carbon isotopic spacing, Δ^13^C_ap-coll_, it is possible to assess whether carbon enriched food sources are protein-depleted or protein-rich. In the case of a monoisotopic diet–that is, when δ^13^C from all macronutrients originates from the same main food source–a spacing of ca. 8.4‰ (13.4–5‰) can be assumed between carbon in dentine and in enamel (Δ^13^C_ap-coll_) because (a) the δ^13^C_enamel_ value in tooth apatite is enriched of ca. 13.4 ± 1.0‰ when compared to the actual diet [82, 84, 85] and (b) a difference of ca. 5‰ between the δ^13^C of the diet and the collagen can be estimated if the δ^13^C value of dietary protein and energy are the same [79]. The Δ^13^C_ap-coll_ is expected to be higher than 8.4‰ if the diet is mainly based on C_4_ carbohydrates and C_3_ proteins. On the contrary, a lower spacing is expected if there is an important consumption of C_3_ carbohydrates and marine protein.

**Fig 7:**
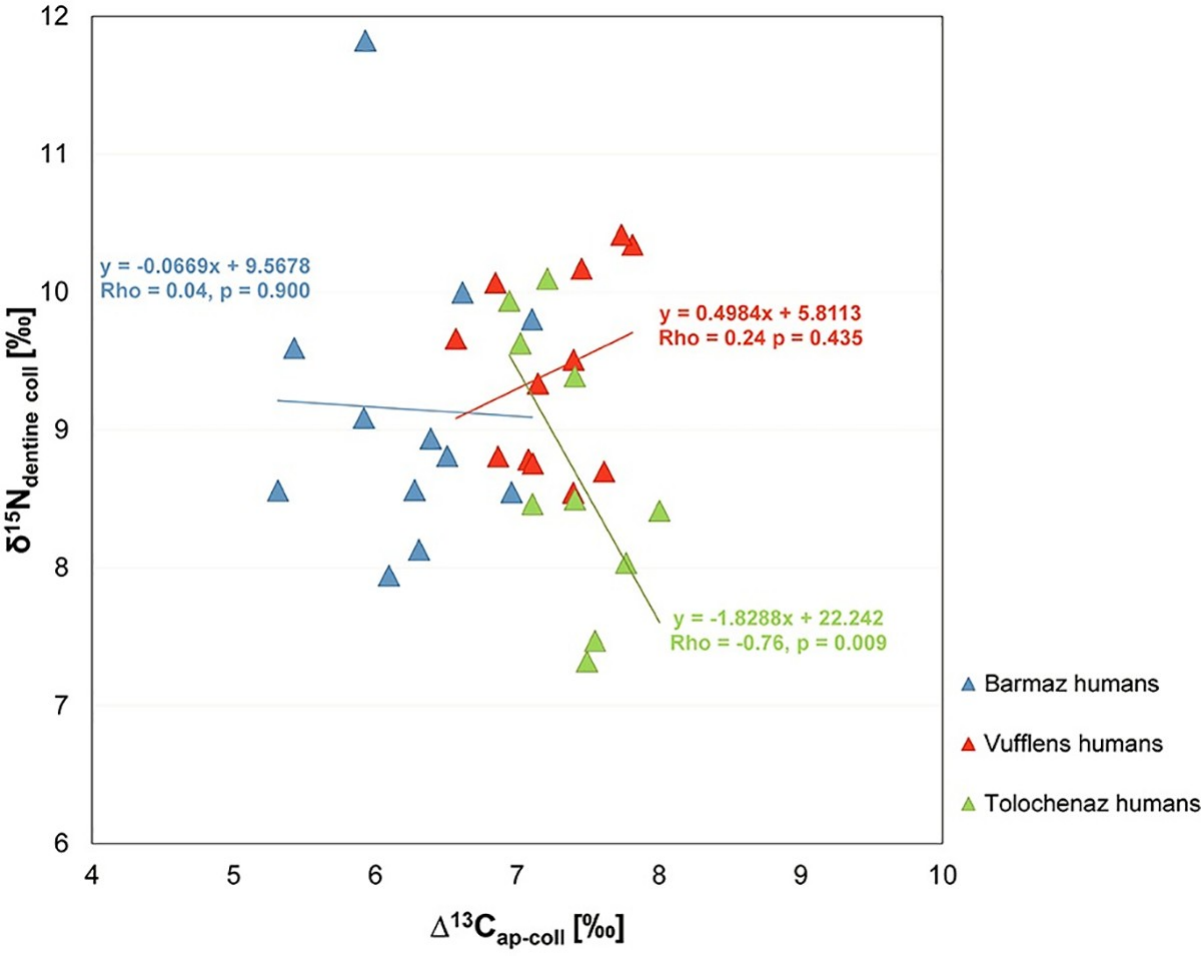
Dentine nitrogen isotope ratios and enamel-dentine collagen spacing for humans from the three sites in western Switzerland.

In this study, the Δ^13^C_ap-coll_ values range from 5.3‰ to 8.0‰. The La Barmaz mean is 6.2‰ ± 0.5‰ (min = 5.3‰, max = 7.1‰). The Vufflens mean is 7.3‰ ± 0.4‰ (min = 6.6‰ max = 7.8‰). Finally, the Tolochenaz mean is 7.4‰ ± 0.7‰ (min = 6.9‰ max = 8.0‰). According to these results, C_3_ plants and animal proteins were consumed in greater proportion during childhood at the beginning of the Bronze Age, whereas the intake of C_4_ plants increased during the later phases of the Bronze Age at the expense of animal protein. This result is confirmed when combining the Δ^13^C_ap-coll_ with the δ^15^N in dentine collagen. Indeed, the results suggest a diet mainly composed of C_3_ protein foods and C_3_ energy food sources in the Early and Middle Bronze Age. During the Final Bronze Age, the main protein intake came from a C_3_ environment but the bulk of the diet included low-protein C_4_ foodstuffs (Fig 7). Furthermore, no correlations occur between the Δ^13^C_ap-coll_ spacing and the dentine δ^15^N values in La Barmaz and Vufflens. However, in Tolochenaz a negative significant correlation is evident suggesting the presence of a strong trophic level effect that varies with the individuals (Fig 7).

In order to evaluate the changes in C_4_ contributions during the Bronze Age, the model elaborated by [161] and [86, 157] was applied (%C_4_ = (−25 − (δ^13^C_bone apatite_ − 9.4)) / 15 × 100). However, this model was developed from experimental studies on δ^13^C_bone apatite_ and δ^13^C_bone collagen_. Tooth enamel apatite ratios are not equivalent to bone apatite ratios [82, 162], therefore, the δ^13^C_enamel_ values were adjusted. Some studies have estimated the offsets between δ^13^C_bone apatite_ and δ^13^C_enamel_ for the same individual in a human population [85, 162–164]. Then, based on these results, an average value of + 1.8‰ has been added to δ^13^C_enamel_ values [165] before calculating the proportion of C_4_ resources consumed. Although the results should be considered with caution due to analytical uncertainty and to inter-individual variability in the estimated enamel to bone spacing, they suggest a broad difference in the proportion of C_4_ plants consumed during childhood by individuals buried at La Barmaz and Vufflens (respectively an estimate of ca. 20 and 26%) and by individuals buried at Tolochenaz (almost half of the diet derived from C_4_ plants, ca. 47%). Thus, δ^13^C_enamel_ value confirms first that the dietary patterns detected in adulthood throughout the Bronze Age are similar to those in childhood and, second, that C_4_ plants were directly consumed by humans.

## Conclusions

This paper presents for the first time a complex multi-isotopic and bioarchaeological study on Bronze Age western Switzerland. This area is a natural and economic crossroads between central Europe and the Mediterranean area and this study contributes to the reconstruction of human dietary changes during the Bronze Age and sheds light on the complexity of human behavior, filling a gap for central Europe.

The botanical data suggest an increased impact of anthropic activities during the Bronze Age. The manuring practices seemed to play an important role in the farming systems, particularly at the end of the Bronze Age. There was greater diversity in cereal cultivation, highlighted by the adoption of new staple crops such as millet varieties. Moreover, plant isotopic data indicate that they grew in good hydric conditions, likely directly managed by humans. As for domestic herbivores, no different herding strategies have been detected throughout the Bronze Age. The fodder they consumed was based on wild plants and/or non-fertilized cultivated plants. Based on a reliable isotopic baseline of coeval local food sources, the human data show that dietary habits changed during the Bronze Age. The staple crops were wheat, barley and pulses during the first phases of the Bronze Age, whilst millets, better adapted to poor soil conditions, were adopted at the end of the Bronze Age. It could be the result of several factors. Among others, exchanges with communities in southern Europe where these crops were already present, like northern Italy, the increasing in dryness caused by the overexploitation of the soils, without excluding the impact of climatic fluctuation along the Bronze Age. Furthermore, there is less animal protein intake during the Final Bronze Age than in previous periods. It is likely that the demographic increase recorded during this phase led to a change in economic strategies, favoring crop cultivation instead of animal husbandry. Further investigations on Final Bronze Age communities could provide more details on this trend.

The comparison between teeth and bone data suggests that, even though a general greater consumption of animal products in childhood than in adulthood is generally highlighted, the main patterns identified in adults are confirmed in juveniles. Thus, the general food habits were not age dependent, but rather, were characteristic of each community. However, some exceptions are present and these individuals could have had a different origin and therefore different dietary habits linked to their local origins.

This research highlights the importance studying the entire trophic chain, including archaeobotanical samples that are still rare in European isotopic research, because this provides crucial details for the reconstruction of the paleoenvironment, agricultural strategies and human dietary choices. Furthermore, multi-sampling analysis of human remains enabling cross-sectional evaluation facilitates tracking dietary changes throughout an individual’s lifespan. This study is the first step to understand how protohistoric populations adapted and modified the landscape where they lived in a limited but well-defined area at the heart of Europe.

## Supporting information

S1 Text **Table A.** Botanical and animal remains analyzed in this study. **Table B.** Bone and dentine collagen and enamel apatite Wilcoxon Mann-Whitney exact text; **Table C.** Bone and dentin collagen Wilcoxon Mann-Whitney exact text according to biological parameters (age and sex); **Table D.** Offsets calculated between plants, human and animal bone collagen.
(DOCX)

S1 Table Bayesian modelling of dietary patterns using FRUITS software. Data entry and results.
(XLSX)

S1 Fig Collombey-Muraz, La Barmaz map of the cemetery with the individuals analyzed (designed by M. Honegger). (TIF)

S2 Fig Vufflens-la-Ville, En Sency: Map of the cemetery with the individuals analyzed (Reprinted but modified from [31] under a CC BY license, with permission from [CAR], original copyright [2005]). (TIF)

S3 Fig Tolochenaz, La Caroline: Map of the cemetery with the individuals analyzed, LaC (Reprinted but modified from [35] under a CC BY license, with permission from [CAR], original copyright [2019]). (TIF)

S4 Fig Box-plot showing the δ^13^C_enamel_ values for Collombey-Muraz, La Barmaz, Vufflens-la-Ville, En Sency and Tolochenaz, Le Boiron, La Caroline humans. (TIF)

S5 Fig Box-plots showing the stable isotope bone and teeth results for the δ^13^C_coll_, δ^15^N_coll._ for Collombey-Muraz, La Barmaz, Vufflens-la-Ville, En Sency and Tolochenaz, Le Boiron, La Caroline humans. (TIF)

S1 File (PDF)
