# Supplementary material for: Bronze Age innovations and impact on human diet: A multi-isotopic and multi-proxy study of western Switzerland
Source: PLoS One. 2021 Jan 27;16(1):e0245726. doi: 10.1371/journal.pone.0245726 (PMC7840060; doi:10.1371/journal.pone.0245726)
Supplement: S1 Text — Table A. Botanical and animal remains analyzed in this study. Table B. Bone and dentine collagen and enamel apatite Wilcoxon Mann-Whitney exact text; Table C. Bone and dentin collagen Wilcoxon Mann-Whitney exact text according to biological parameters (age and sex); Table D. Offsets calculated between plants, human and animal bone collagen. (DOCX) [file pone.0245726.s001.docx]

**S1 Text (tables)**

**Table A. Botanical and animal remains analysed in this study.**

| **Sites** | **Period** | **Species** | **N** |
| --- | --- | --- | --- |
| **Collombey-Muraz, La Barmaz** | Early Bronze Age  (2200-1500 BCE) | 2 *Ovis aries/Capra hircus*  3 *Bos taurus*  2 *Sus domesticus* | 7 |
| **Rances, Champ Vully** | Middle Bronze Age  (1550-1300 BCE) | 1 *Bos taurus*  1 *Sus domesticus* | 2 |
| **Chindrieux** | Final Bronze Age  (1050-800 BCE) | 4 *Esox lucius* | 4 |
| **Chens-sur-Léman, Tougues** | Final Bronze Age  (1050-800 BCE) | 2 *Ovis aries*  *1 Capra hircus*  2 *Bos taurus*  3 *Sus domesticus*  1 *Cervus elaphus* | 9 |
| **TOTAL** | | | **22** |
| **Chens-sur-Léman,**  **Pré d’Ancy and Vereître** | Early Bronze Age  (2200-1500 BCE) | 3 *Triticum* sp. | 3 |
|  | Middle-Final Bronze Age  (1500-950 BCE) | 1 *Vicia faba*  2 Fabacea  3 *Hordeum vulgare*  *1 Setaria italica* | 7 |
|  | Final Bronze Age  (950-800 BCE) | 4 *Vicia* sp.  1 Fabacea  2 *Triticum monococcum/dicoccum*  4 *Triticum dicoccum*  3 *Triticum* sp.  1 *Hordeum vulgare*  1 *Setaria italica*  2 *Panicum miliaceum*  2 *Setaria/Panicum* | 20 |
| **TOTAL** | | | **30** |

**Table B. Bone and dentine collagen and enamel apatite Wilcoxon Mann-Whitney exact text.**

|  | **Bone** | | | **Teeth** | | |
| --- | --- | --- | --- | --- | --- | --- |
|  | **Collombey-Muraz,**  **La Barmaz** | **Vufflens-la-Ville,**  **En Sency** | **Tolochenaz,**  **Le Boiron, La Caroline** | **Collombey-Muraz,**  **La Barmaz** | **Vufflens-la-Ville,**  **En Sency** | **Tolochenaz,**  **Le Boiron, La Caroline** |
| **Collombey-Muraz,**  **La Barmaz** | **-** | δ^13^C_coll,_ *p* = 0.468  **δ^15^N_,_ *p* = 0.040**  **δ ^34^S_,_ *p* = 0.009** | **δ^13^C_coll,_ *p* < 0.001**  δ^15^N_,_ *p* = 0.0738  **δ ^34^S_,_ *p* = 0.004** | **-** | δ^13^C_coll,_ *p* = 0.296  δ^15^N_,_ *p* = 0.234  **δ^13^C_enamel,_ *p* < 0.001** | **δ^13^C_coll,_ *p* < 0.001**  δ^15^N_,_ *p* = 0.354  **δ^13^C_enamel,_ *p* < 0.001** |
| **Vufflens-la-Ville,**  **En Sency** | δ^13^C_coll,_ *p* = 0.468  **δ^15^N_,_ *p* = 0.040**  **δ ^34^S_,_ *p* = 0.009** | **-** | **δ^13^C_coll,_ *p* < 0.001**  **δ^15^N_,_ *p* < 0.001**  δ ^34^S_,_ *p* = 0.121 | δ^13^C_coll,_ *p* = 0.296  δ^15^N_,_ *p* = 0.234  **δ^13^C_enamel,_ *p* < 0.001** | **-** | **δ^13^C_coll,_ *p* < 0.001**  δ^15^N_,_ *p* = 0.051  **δ^13^C_enamel,_ *p* < 0.001** |
| **Tolochenaz,**  **Le Boiron, La Caroline** | **δ^13^C_coll,_ *p* < 0.001**  δ^15^N_,_ *p* = 0.073  **δ ^34^S_,_ *p* = 0.004** | **δ^13^C_coll,_ *p* < 0.001**  **δ^15^N_,_ *p* < 0.001**  δ ^34^S_,_ *p* = 0.121 | **-** | **δ^13^C_coll,_ *p* < 0.001**  δ^15^N_,_ *p* = 0.354  **δ^13^C_enamel,_ *p* < 0.001** | **δ^13^C_coll,_ *p* < 0.001**  δ^15^N_,_ *p* = 0.051  **δ^13^C_enamel,_ *p* < 0.001** | **-** |

**Table C. Bone and dentin collagen Wilcoxon Mann-Whitney exact text according to biological parameters (age and sex).**

Age group: 5-15 child (C); 16-20 adolescent (a); 20-30 young adult (YA); > 30 adult (A).

| **SEX** | | | | **AGE** | |
| --- | --- | --- | --- | --- | --- |
| **bone** | | **teeth** | | **bone** | **teeth** |
| **Collombey-Muraz,**  **La Barmaz** | **Vufflens-la-Ville,**  **En Sency** | **Collombey-Muraz,**  **La Barmaz** | **Vufflens-la-Ville,**  **En Sency** | **Vufflens-la-Ville**  **(Adults = A + YA, Juveniles = a+C)** | **Vufflens-la-Ville**  **(Adults = a + YA, Juveniles = a+C)** |
| δ^13^C_coll_ *p* = 0.116  δ^15^N_,_ *p* = 0.416  δ ^34^S_,_ *p* = 0.064 | δ^13^C_coll_ *p* = 0.873  δ^15^N_,_ *p* = 1  δ ^34^S_,_ *p* = 0.682 | δ^13^C_coll_ *p* = 0.179  δ^15^N_,_ *p* = 0.789  δ^13^C_enamel,_ *p* = 0.253 | δ^13^C_coll_ *p* = 0.214  δ^15^N_,_ *p* = 1  δ^13^C_enamel,_ *p* = 0.428 | δ^13^C_coll_ *p* = 0.819  δ^15^N_,_ *p* = 0.292  δ ^34^S_,_ *p* = 0.915 | δ^13^C_coll_ *p* = 0.281  δ^15^N_,_ *p* = 1  δ^13^C_enamel,_ *p* = 0.367 |

**Table D. Offsets calculated between plants, human and animal bone collagen.**

| **Collombey-Muraz, La Barmaz**^a^ | | **Vufflens-la-Ville, En Sency** ^b^ | | **Tolochenaz, Le Boiron, La Caroline** ^c^ | |
| --- | --- | --- | --- | --- | --- |
| ∆^13^C_animals-plants_ = 4.7‰ | ∆^15^N_animals-plants_ = 1.3‰ | ∆^13^C_animals-plants_ = 3.6‰ | ∆^15^N_animals-plants_ = 2.2‰ | ∆^13^C_animals-plants_ = 2.9‰ | ∆^15^N_animals-plants_ = 1.6‰ |
| ∆^13^C_humans-plants_ = 5.2‰ | ∆^15^N_humans-plants_ = 5‰ | ∆^13^C_humans-plants_ = 4.3‰ | ∆^15^N_humans-plants_ = 5.9‰ | ∆^13^C_humans-plants_= 5.8‰ | ∆^15^N_humans-plants_ = 4.1‰ |
| ∆^13^C_humans- animals_ = 0.5‰ | ∆^15^N_humans- animals_ = 3.7‰ | ∆^13^C_humans- animals_ = 0.7‰ | ∆^15^N_humans- animals_= 3.7‰ | ∆^13^C_humans- animals_= 3‰ | ∆^15^N_humans-animals_ = 2.5‰ |

^a^plants: EBA C_3_ plants (wheat and barley when present); animals: herbivores and pigs with herbivore diet

^b^plants: MBA-FBA C_3_ plants (wheat and barley when present); animals: because of the small sample size of MBA animals and no significant differences occur among BA animals, we refer to all BA herbivores and pigs with herbivore diet

^c^plants: FBA C_3_ plants (wheat and barley when present); animals: herbivores and pigs with herbivore diet
