# Supplementary figures and images for: Bronze Age innovations and impact on human diet: A multi-isotopic and multi-proxy study of western Switzerland
Source: PLoS One. 2021 Jan 27;16(1):e0245726. doi: 10.1371/journal.pone.0245726 (PMC7840060; doi:10.1371/journal.pone.0245726)

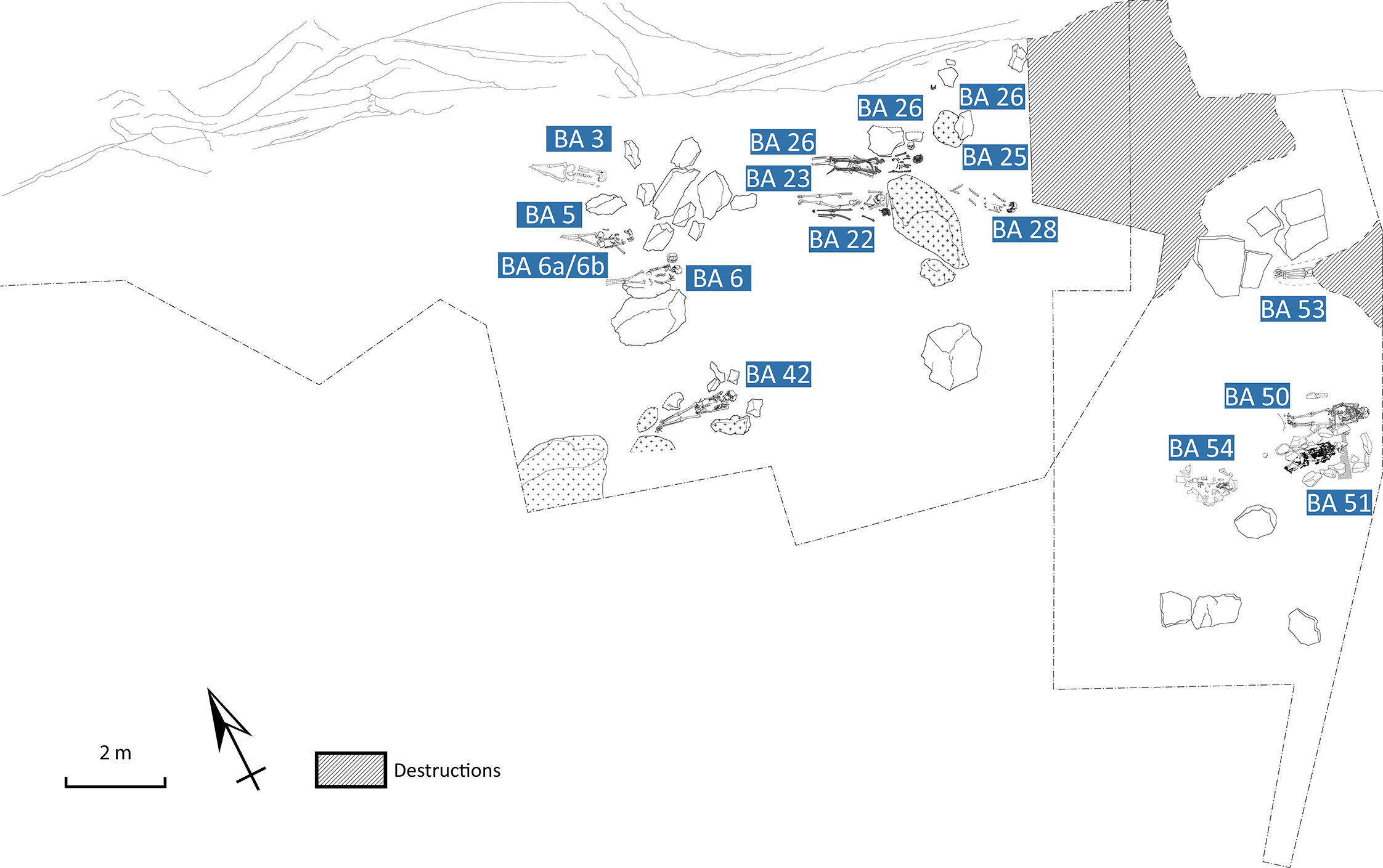

Supplement: S1 Fig — (TIF) [file pone.0245726.s003.tif]

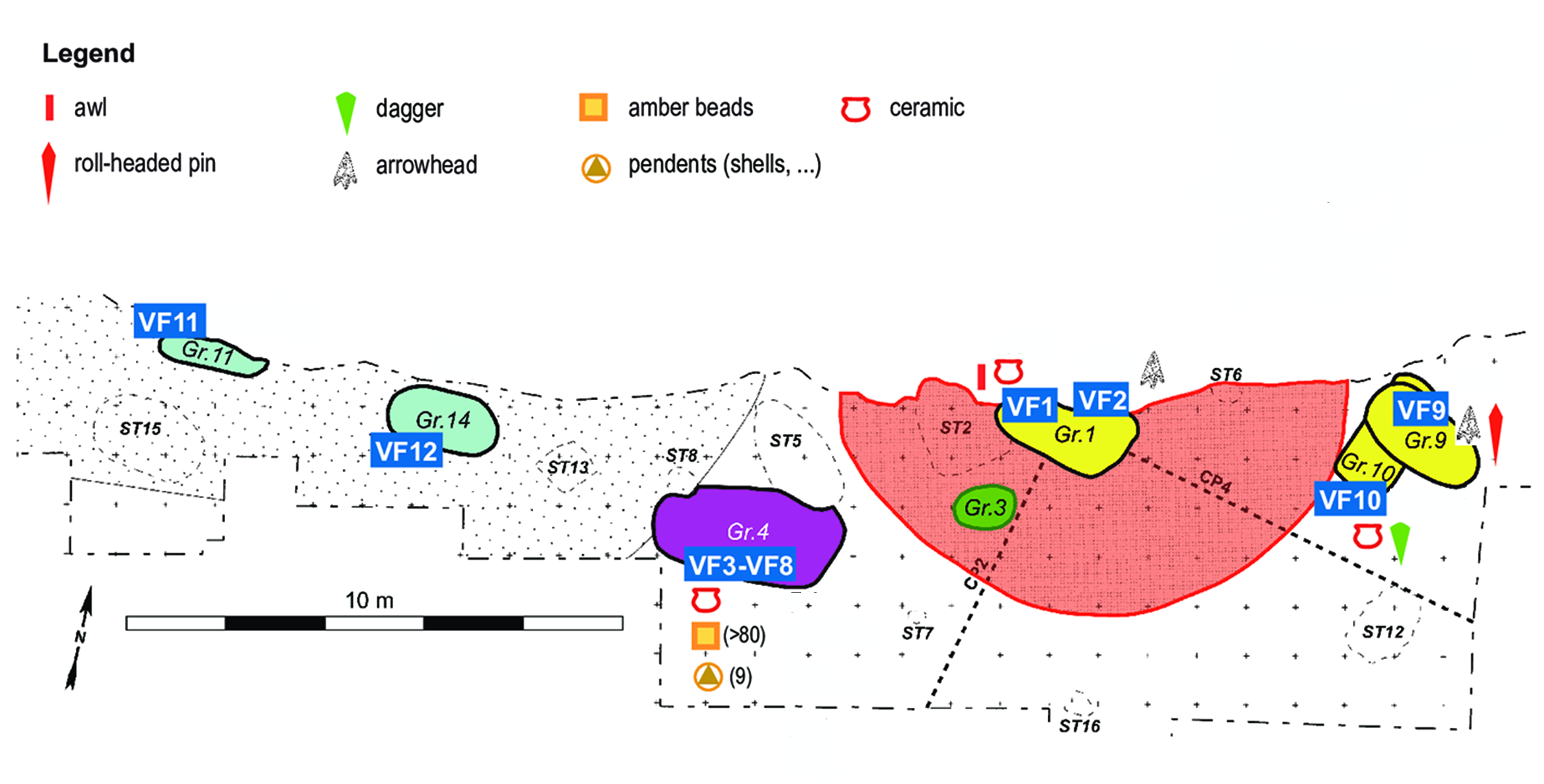

Supplement: S2 Fig — (TIF) [file pone.0245726.s004.tif]

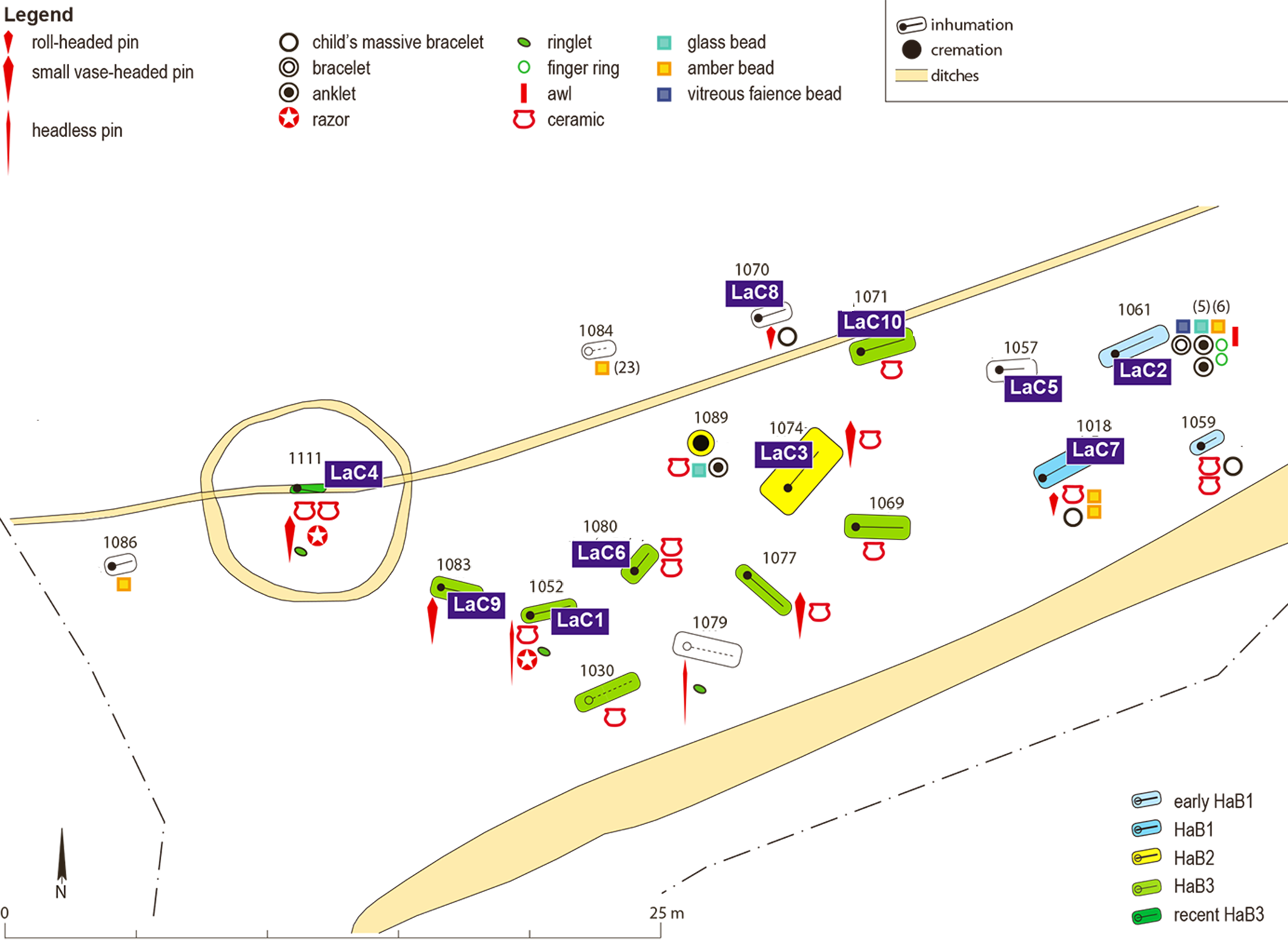

Supplement: S3 Fig — (TIF) [file pone.0245726.s005.tif]

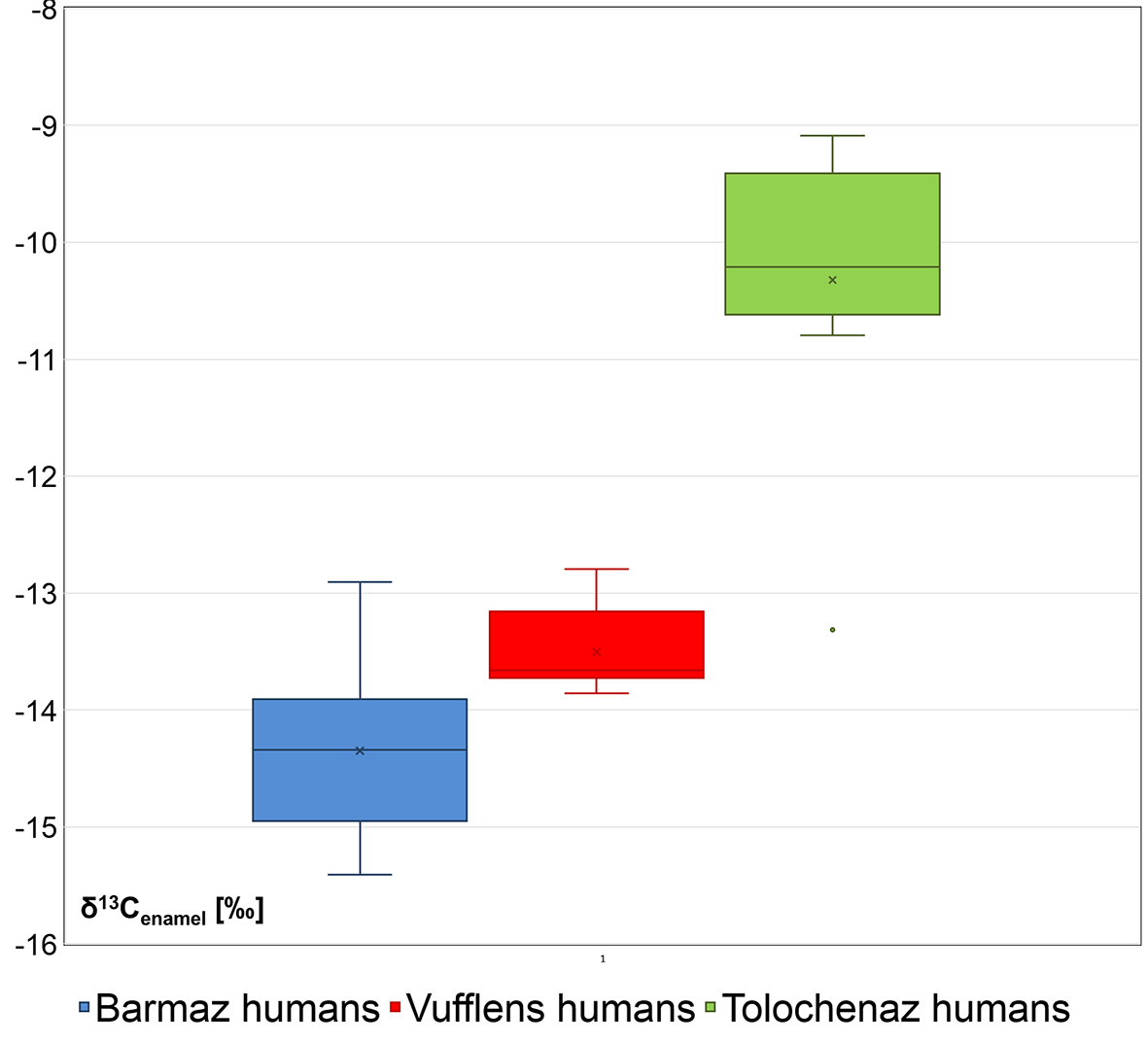

Supplement: S4 Fig — (TIF) [file pone.0245726.s006.tif]

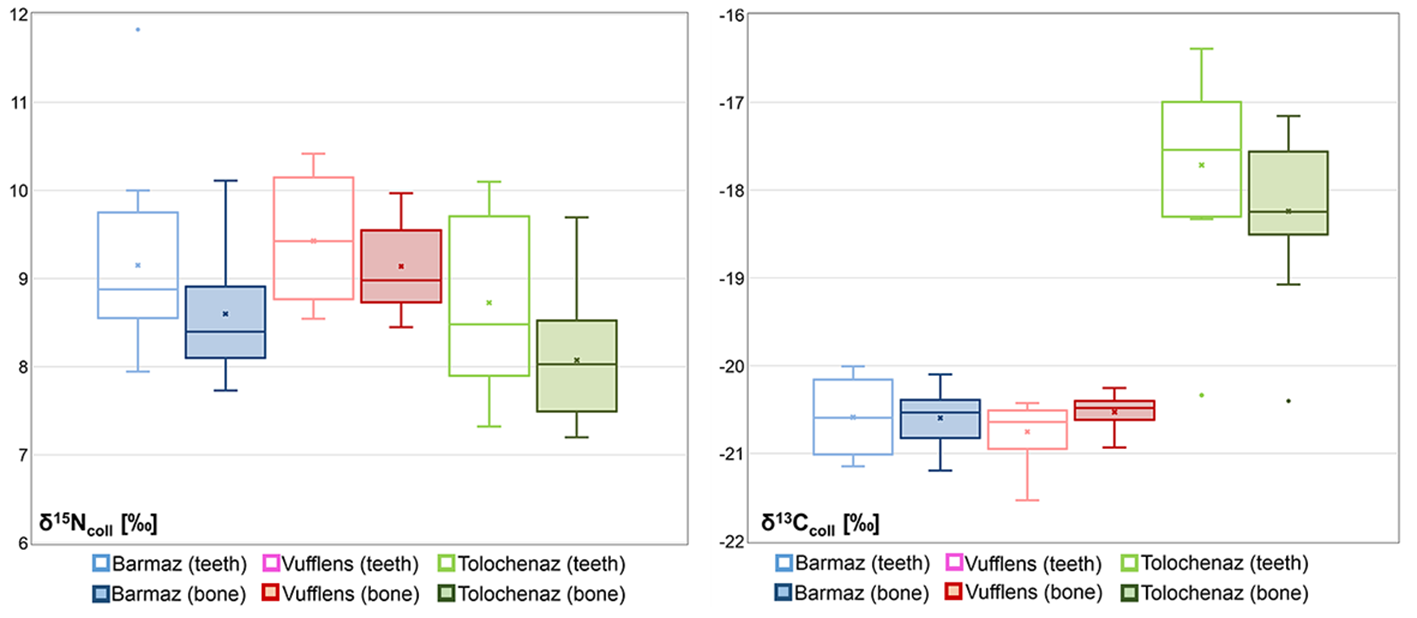

Supplement: S5 Fig — (TIF) [file pone.0245726.s007.tif]
