# Supplementary material for: Bronze Age innovations and impact on human diet: A multi-isotopic and multi-proxy study of western Switzerland
Source: PLoS One. 2021 Jan 27;16(1):e0245726. doi: 10.1371/journal.pone.0245726 (PMC7840060; doi:10.1371/journal.pone.0245726)
Supplement: S1 File — (PDF) [file pone.0245726.s008.pdf]

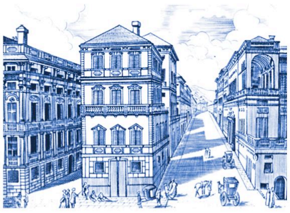

UNIVERSITÀ DEGLI STUDI DI GENOVA

D.A.F I.S T.

DIPARTIMENTO DI ANTICHITÀ, FILOSOFIA, STORIA

Via Balbi, 2 - 4 - 6 16126 Genova

July 16, 2020

Dear Dr Varalli,

I am pleased to confirm that your article

Alessandra Varalli et al. : Ricostruzione dei pattern alimentari e delle pratiche di allattamento e svezzamento dal Neolitico alle età dei metalli tramite analisi multi-isotopiche della dentina incrementale e del collagene osseo

has been accepted for publication and will be included in the following volume which is in the final stages of preparation by:

Fabio Negrino, Vincenzo Tiné (Eds) - LIII Scientific Meeting of the Italian Institut for Prehistory and Protohistory. Prehistory and Protohistory In Liguria. Rivista di Scienze Preistoriche (2020).

I am expecting the submission of the final manuscript very soon. Publication will then follow quickly.

Publication will be in print and e-format.

Yours sincerely

Prof. Fabio Negrino

Editor of the volume

DAFIST – Department of Antiquities, Philosophy and History
University of Genoa
Via Balbi, 2 – I-16126 Genova (Italy)
e-mail: fabio.negrino@unige.it
Tel. (0039)-010-209-51803
Tel. (0039)-010-209-59712 (Laboratorio)
Mobile (0039)-347-5349133
